# Substituted arylsulphonamides as inhibitors of perforin-mediated lysis

**DOI:** 10.1016/j.ejmech.2017.05.048

**Published:** 2017-09-08

**Authors:** Julie A. Spicer, Christian K. Miller, Patrick D. O'Connor, Jiney Jose, Kristiina M. Huttunen, Jagdish K. Jaiswal, William A. Denny, Hedieh Akhlaghi, Kylie A. Browne, Joseph A. Trapani

**Affiliations:** a Auckland Cancer Society Research Centre, Faculty of Medical and Health Sciences, The University of Auckland, Private Bag 92019, Auckland 1142, New Zealand; b Maurice Wilkins Centre for Molecular Biodiscovery, A New Zealand Centre for Research Excellence, Auckland, New Zealand; c School of Pharmacy, Faculty of Health Sciences, University of Eastern Finland, P.O. Box 1627, FI-70211 Kuopio, Finland; d Cancer Immunology Program, Peter MacCallum Cancer Centre, 305 Grattan Street, Melbourne, Victoria 3000, Australia; e Sir Peter MacCallum Department of Oncology, The University of Melbourne, Parkville, Victoria 3052 Australia

**Keywords:** Perforin, Perforin inhibitor, Arylsulphonamide, Bioisostere, Immunosuppressant

## Abstract

The structure-activity relationships for a series of arylsulphonamide-based inhibitors of the pore-forming protein perforin have been explored. Perforin is a key component of the human immune response, however inappropriate activity has also been implicated in certain auto-immune and therapy-induced conditions such as allograft rejection and graft versus host disease. Since perforin is expressed exclusively by cells of the immune system, inhibition of this protein would be a highly selective strategy for the immunosuppressive treatment of these disorders. Compounds from this series were demonstrated to be potent inhibitors of the lytic action of both isolated recombinant perforin and perforin secreted by natural killer cells *in vitro*. Several potent and soluble examples were assessed for *in vivo* pharmacokinetic properties and found to be suitable for progression to an *in vivo* model of transplant rejection.

## Highlights

• A new series of arylsulphonamides that inhibit perforin have been identified.
• Potency against both recombinant perforin and whole NK cells was optimised.
• *In vitro* toxicity, solubility and stability was measured for selected candidates.
• *In vivo* PK was determined to select potential candidates for efficacy studies.
• Several compounds were selected for evaluation in a mouse transplant rejection model.

## Graphical abstract

**Figure:**
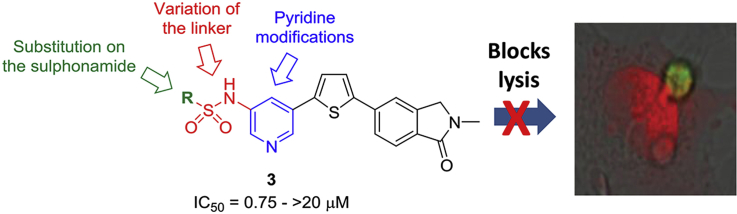

## 1 Introduction

Perforin is a 67 kDa, calcium-dependent glycoprotein expressed by only the natural killer (NK) cells and cytotoxic T lymphocytes (CTLs) of the mammalian immune system [1], [2]. These “killer lymphocytes” utilise the pore-forming ability of perforin as a critical component of the granule exocytosis pathway; the principal mechanism used by NK and CTL cells for tumour immunosurveillance and as a defence against viral infection and intracellular pathogens [3]. Identification of a target cell by an effector cell results in the formation of an immune synapse whereupon CTL (or NK) secretory granules polarise to the site of contact. These granules contain both perforin and a group of pro-apoptotic serine proteases known as granzymes, and upon fusion with the CTL plasma membrane, release their luminal contents into the synapse [2], [4]. Perforin performs a key role in this process because entry of the granzymes required for cell death into the target cell cytosol is solely dependent on its presence [1], [5].

Although perforin is synthesized and secreted into the immune synapse as a monomer, it rapidly binds to the target cell membrane through its calcium-dependent C2 domain [6], [7] and oligomerises into large transmembrane pores composed of approximately 24 perforin monomers. This process was elucidated using a combination of the perforin X-ray crystal structure and cryoelectron microscopy to reconstruct an entire perforin pore [8]. Electron microscopy, X-ray crystallography and functional studies have also shown that the process involves electrostatic interactions which include a salt bridge formed between R213 on the ‘front’ surface of one monomer interacting with E343 on the ‘back’ surface of the adjacent monomer [9]. Similarly, mutational studies reveal that D191, which is immediately adjacent to R213, makes interactions that are key to oligomerisation and that substitution with a bulky hydrophobic residue (D191V) abrogates this process [9].

Until recently, the precise mechanism of granzyme entry into the target cell was debated, but it is beyond any doubt that the pore-forming activity of perforin is indispensable. In essence, secreted perforin forms large (18 nm diameter) transmembrane pores on the surface of the target cell, through which the granzyme monomers (4 nm diameter) diffuse into the cytosol [10], [11]. Once internalised the granzymes cleave key substrates to initiate rapid apoptotic death [5], [10], [11], [12], [13]. Unlike the granzymes, which are encoded by many genes and are, therefore, subject to considerable redundancy of function, the gene encoding perforin (PRF1) is present as a single copy in all mammals. Gene-targeting studies in mice [1] and naturally occurring disease-causing mutations in humans [14], [15] confirm that perforin deficiency cannot be compensated by any other protein. This makes perforin an ideal target for therapeutic intervention.

While perforin is a key component of the immune response, inappropriate activity has also been implicated in a number of human immunopathologies and therapy-induced conditions. These include cerebral malaria, insulin-dependent diabetes, juvenile idiopathic arthritis and postviral myocarditis [16], [17], [18], as well as therapy-induced conditions such as allograft rejection and graft versus host disease [19], [20], [21]. Our current goal is to seek small molecule inhibitors of perforin as potential immunosuppressive agents for the treatment of autoimmune diseases and other conditions characterised by dysfunction of this pathway. This should be a highly selective strategy since perforin is expressed exclusively by CTL and NK cells, in contrast to approaches using conventional immunosuppression treatments which indiscriminately depress immune function [22], [23], [24].

**Fig. 1:**
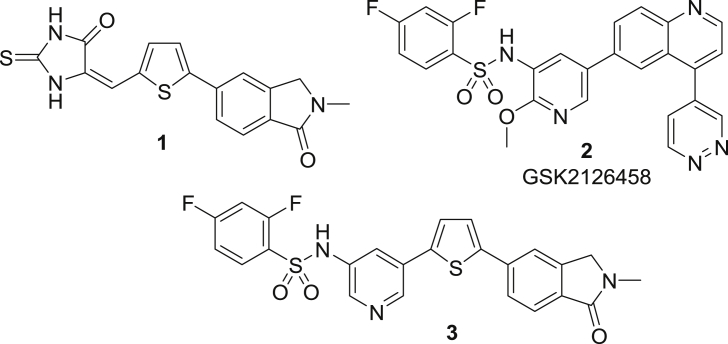
Perforin inhibitors and PI3Kα clinical candidate GSK2126458.

Based on an initial hit from a mass screen [25], we have previously designed and optimised inhibitors of perforin that can; (i) block recombinant purified perforin, (ii) block perforin delivered by intact NK cells and, (iii) withstand incubation in serum (e.g. **1**; Fig. 1) [26], [27], [28], [29], [30].

**Fig. 2:**
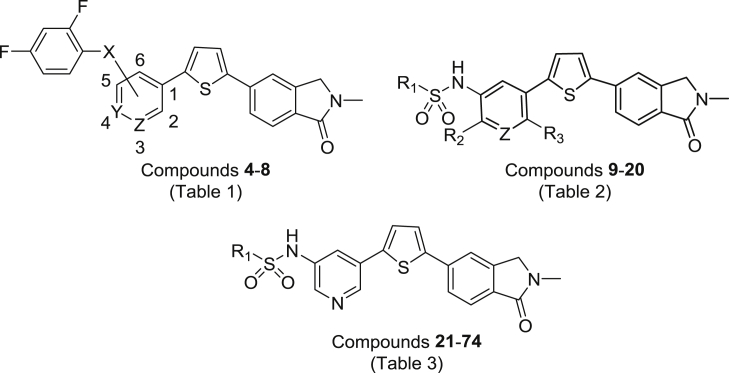
Variations on **3** to give new benzenesulphonamide analogues.

While these compounds appeared highly promising, replacement of the 2-thioxoimidazolidinone moiety that contained a potential Michael acceptor and showed variable toxicity toward perforin-producing NK cells proved problematic. This issue was only overcome when we amalgamated our own finding that an aryl sulphonamide could act as a bioisosteric replacement [30] with a strategy implemented by GSK workers, where a thiazolidinedione was replaced with a pyridyl-linked benzenesulphonamide to give **2** [31]. This approach resulted in a new series of benzenesulphonamide-based perforin inhibitors, exemplified by **3**, which were potent, soluble and essentially non-toxic toward NK cells [32]. In the following report we extend our study to explore whether it is possible to further modulate activity and physicochemical properties through variation of the sulphonamide linker, linker position, and substitution on the central pyridine ring and terminal benzene (Fig. 2).

## 2 Results and discussion

### 2.1 Chemistry

**Scheme 1:**
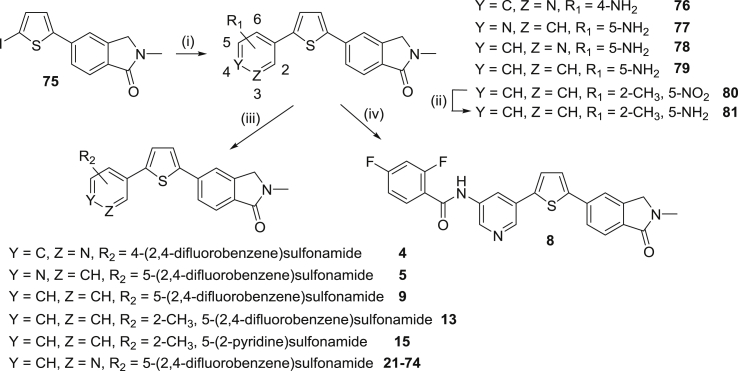
Reagents and conditions: (i) Boronate, Pd(dppf)Cl_2_, EtOH/toluene, 2 M Na_2_CO_3_, reflux; (ii) H_2_, 60 psi, 1:1:1 EtOH/EtOAc/THF, RT, 3 h; (iii) 2,4-Difluorobenzenesulfonyl chloride or pyridine-2-sulfonyl chloride, pyridine, 0–45 °C; (iv) 2,4-Difluorobenzenoic acid chloride, pyridine, 0–45 °C, 16 h.

The majority of the target compounds were constructed from right to left starting with our previously published key iodide **75** [32] (Scheme 1). Suzuki reaction of **75** with a variety of commercially available aminopyridine boronates under standard conditions gave the required amine intermediates **76**–**79** which were subsequently reacted with a range of substituted aryl sulphonyl chlorides. The 5-amino-3-pyridine derivative **78** [32] in particular was employed in the preparation of all compounds in Table 3. One exception was where the central pyridine ring was replaced with a benzene; in this case the Suzuki step was carried out with 2-methyl-5-nitrobenzeneboronic acid, the nitro compound (**80**) hydrogenated to give the amine (**81**), which was then reacted with either 2,4-difluorobenzenesulphonyl chloride or 2-pyridinesulphonyl chloride to afford **13** and **15** respectively. Finally, amido-linked compound **8** was prepared by reaction of **78** with 2,4-difluorobenzoic acid chloride.

**Scheme 2:**
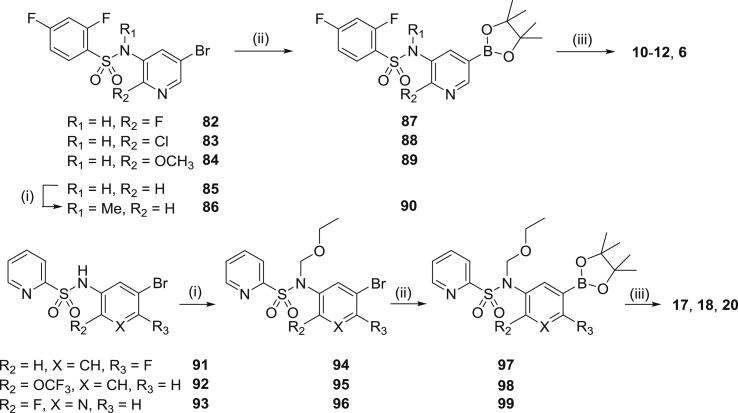
Reagents and conditions: (i) a. NaH, DMF, 0° C, 0.5 h, b. MeI or (chloromethoxy)-ethane, DMF, 0° C-RT, 1.5 h; (ii) Bis(pinacolato)diboron, KOAc, Pd(dppf)Cl_2_, DMSO, 90 °C, 3 h.; (iii) a. **75**, Pd(dppf)Cl_2_, EtOH/toluene, 2 M Na_2_CO_3_, reflux, b. Deprotection if required: 3 M HCl/1,4-dioxane (1:1), 1 h.

In a smaller number of cases, mostly those examples with substitution on the central pyridine ring, the target compounds were effectively synthesized from two halves; the fully elaborated left-hand side benzenesulphonamide subunit (e.g **82**–**85**) and key iodide **75** as the right-hand side (Scheme 2). The intermediate bromides **82**–**85** and **91**–**93** were prepared from a variety of commercially available aryl sulphonyl chlorides and substituted 3-aminopyridines (or anilines) under standard conditions. In the case of **85**, the sulphonamide NH was methylated with NaH and MeI in DMF to give **86**, and for **91**–**93** where protection of this NH was required for the subsequent coupling to be successful, the alkylation was carried out with (chloromethoxy)ethane to give **94**–**96**. All bromides were converted to the corresponding boronates **87**–**90** and **97**–**99** under palladium-catalysed conditions using bis(pinacolato)diboron and KOAc in DMSO, then finally reacted in a Suzuki step with iodide **75** to introduce the thiophene-*N*-methylisoindolinone subunit (**6**, **10**–**12**, **17**, **18**, **20**). Where required (for **17**, **18**, **20**), deprotection was carried out under acidic conditions.

**Scheme 3:**
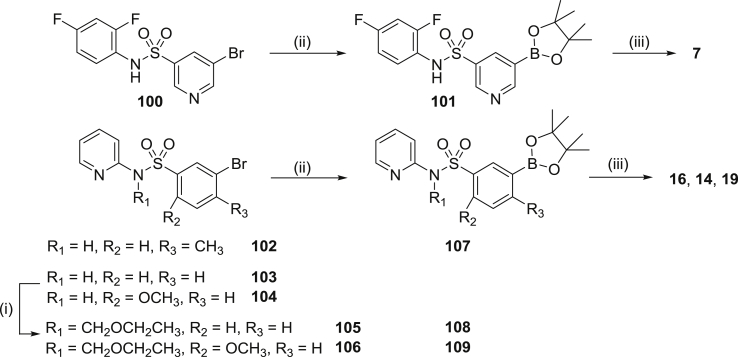
Reagents and conditions: (i) a. NaH, DMF, 0 °C, 0.5 h, b. (Chloromethoxy)ethane, DMF, 0 °C-RT, 1.5 h; (ii) Bis(pinacolato)diboron, KOAc, Pd(dppf)Cl_2_, DMSO, 90 °C, 3 h.; (iii) a. **75**, Pd(dppf)Cl_2_, EtOH/toluene, 2 M Na_2_CO_3_, reflux, b. Deprotection if required: 3 M HCl/1,4-dioxane (1:1), 1 h.

A limited number of “reverse” sulphonamides were also prepared (Scheme 3). In the case of target compound **7**, intermediate bromide **100** was prepared from 2,4-difluoroaniline and 5-bromopyridine-3-sulphonyl chloride. Protection of the sulphonamide was not required and conversion to the boronate **101** and subsequent Suzuki coupling with **75** to give **7** proceeded smoothly. Likewise, bromide **102** was prepared from 2-aminopyridine 3-bromo-4-methylbenzenesulfonyl chloride, converted to the boronate **107** and therein coupled with **75** to afford the final product **16**. For bromides **103** and **104**, protection of the sulphonamide NH (**105**, **106**) was required for the sequential borylation (**108**, **109**) and Suzuki reactions to proceed in good yield, and afforded targets **14** and **19** respectively in good yield.

### 2.2 Inhibition of recombinant perforin-mediated lysis

In our initial report describing the discovery of benzenesulphonamide-based inhibitors of perforin [32], analogue design focussed on exploration of the thiophene and *N*-methylisoindolinone subunits which comprise the right-hand side of the molecule. For the current study we sought to further optimise potency and physicochemical characteristics through manipulation of the central pyridine and sulphonamide linker, as well as employing a wide range of substituents on the left-hand benzene (or aryl) ring.

Table 1 shows a group of compounds that explore the effect of changing the position of the sulphonamide link to the pyridine, the location of the pyridine nitrogen, and modification/replacement of the sulphonamide itself.

**Table 1:**
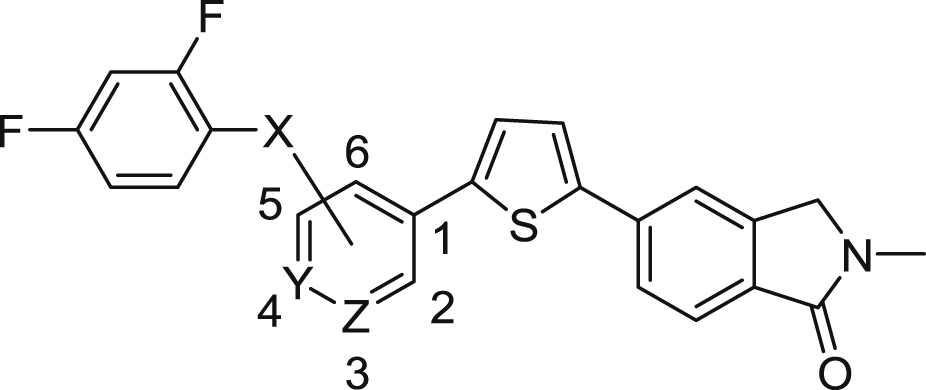
Variation of the sulphonamide linker and position.

Compound	X (linker)	Linker Position	Y	Z	Inhibition of Jurkat Cell Lysis IC_50_ (μM)
**3**^a^	SO_2_NH	5	CH	N	1.17
**4**	SO_2_NH	4	C	N	>20
**5**	SO_2_NH	5	N	CH	4.70
**6**	SO_2_NCH_3_	5	CH	N	16.1
**7**^b^	NHSO_2_	5	CH	N	2.24
**8**	CONH	5	CH	N	>20

a Included as a comparator; see Ref. [32].

b Reverse sulphonamide.

By moving the sulphonamide link from the 5-position (**3**) to the 4-position (**4**), activity is abolished. If the sulphonamide is retained in the preferred 5-position and the pyridine nitrogen moved to the 4-position (**5**), activity is lost 4-fold compared to the lead, **3** (IC_50_s = 4.70 and 1.17 μM respectively). The requirement for a free *N*H in the sulphonamide was probed through methylation of **3** to give *N*-methyl compound **6**. It appears likely that this acidic hydrogen is required for interaction with the target protein since a 14-fold drop in activity to IC_50_ = 16.1 μM was observed. A “reverse” sulphonamide (**7**) is however still acceptable, with less than a 2-fold reduction in activity (IC_50_ = 2.24 μM). Finally, replacement of the sulphonamide with a carboxamide (**8**) results in complete loss of potency, further supporting our hypothesis that the sulphonamide *N*H is required in the linker for optimal activity.

The effect of variation about the central pyridine ring was investigated next (Table 2). Direct replacement of the pyridine (**3**) with a benzene (**9**) resulted in a loss of activity from IC_50_ = 1.17 μM to 5.74 μM. Introduction of a 2-fluoro- (**10**) or 2-chloro-substituent (**11**) to the pyridine gave similar activity to **3** (IC_50_s = 1.03 and 1.99 μM respectively), while the electron-donating 2-methoxy-substituent (**12**) gave an analogue which was somewhat poorer (IC_50_ = 3.56 μM). Benzene-linked compounds (**13**–**19**) were then explored to determine if substitution on the benzene ring could improve activity. Introduction of a single methyl group on **9** to give **13** resulted in complete loss of potency, however at this point we reasoned that the increased lipophilicity and reduced solubility associated with the presence of two benzene rings (**9** and **13**) could be improved if we effectively moved the central pyridine of **3** to the other side of the sulphonamide link (2-pyridylsulphonamide compounds **14**–**20**). This enabled us to explore a small number of compounds containing substitution on the central benzene ring, without further detriment to the overall physicochemical properties. Although far from a comprehensive list, the introduction of methyl (**15**; IC_50_ = 15.4 μM), fluoro (**17**; >20 μM), trifluoromethoxy (**18**; >20 μM) and methoxy (**19**; 9.68 μM) groups proved unsuccessful. The effect of reversing the sulphonamide orientation is consistent with Table 1, appearing to be slightly detrimental, although we only had a single matched pair for this comparison (**15** vs **16**; IC_50_s = 15.4 vs > 20 μM). Lastly, compound **20** contains pyridine rings on either side of the sulphonamide link. While the solubility was significantly improved, the resulting activity (IC_50_ = 4.10 μM) was still no improvement over the lead **3**.

**Table 2:**
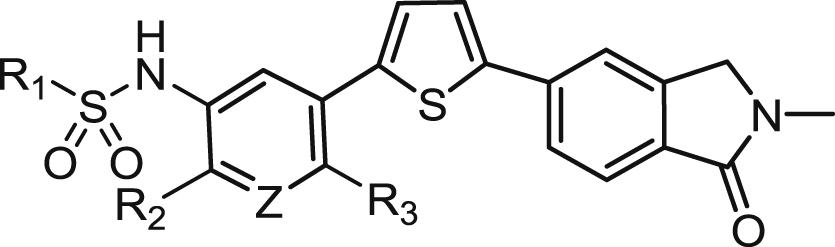
Variation of the pyridine ring.

Compound	R_1_	R_2_	Z	R_3_	Inhibition of Jurkat Cell Lysis IC_50_ (μM)
**3**^a^	2,4-diF-benzene	H	N	H	1.17
**9**	2,4-diF-benzene	H	CH	H	5.74
**10**	2,4-diF-benzene	F	N	H	1.99
**11**	2,4-diF-benzene	Cl	N	H	1.03
**12**	2,4-diF-benzene	OCH_3_	N	H	3.56
**13**	2,4-diF-benzene	H	CH	CH_3_	>20
**14**^b^	2-pyridyl	H	CH	H	8.92
**15**	2-pyridyl	H	CH	CH_3_	15.4
**16**^b^	2-pyridyl	H	CH	CH_3_	>20
**17**	2-pyridyl	H	CH	F	>20
**18**	2-pyridyl	OCF_3_	CH	H	>20
**19**^b^	2-pyridyl	OCH_3_	CH	H	9.68
**20**	2-pyridyl	F	N	H	4.10

a Included as a comparator; see Ref. [32].

b Reverse sulphonamide.

Table 1, Table 2 are limited to either 2,4-difluorobenzene- or 2-pyridyl-sulphonamides. Thus it remained for us to investigate the effect of alternative substituents on the left-hand side of the molecule to determine if we could optimise potency and physicochemical properties further. A total of 52 new substituted benzene or aryl sulphonamides are shown in Table 3.

**Table 3:**
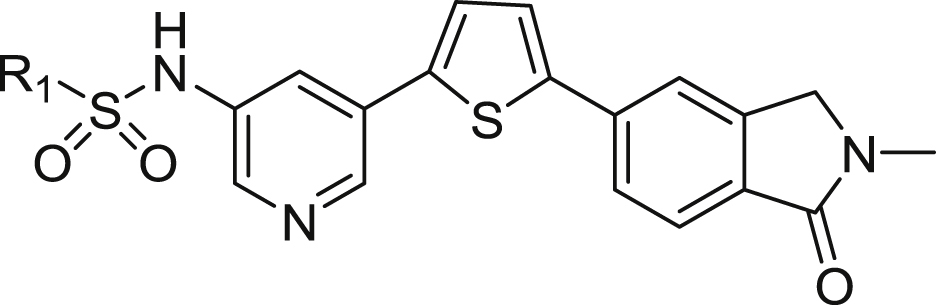
Modulation of activity through substitution on the sulphonamide.

Compound	R_1_	Inhibition of Jurkat Cell Lysis IC_50_ (μM)
**21**	benzene	8.46
**22**	2-F-benzene	2.03
**23**	3-F-benzene	∼20
**24**	4-F-benzene	9.65
**3**^a^	2,4-diF-benzene	1.17
**25**	3,4-diF-benzene	18.6
**26**	2,4,6-triF-benzene	1.76
**27**	2-Cl-benzene	4.01
**28**	3-Cl-benzene	2.42
**29**	4-Cl-benzene	5.39
**30**	3,4-diCl-benzene	1.32
**31**	2,4-diCl-benzene	15.0
**32**	2-Br-benzene	2.66
**33**	3-Br-benzene	2.11
**34**	4-Br-benzene	8.77
**35**	2,4-diBr-benzene	2.87
**36**	4-I-benzene	5.00
**37**	2-OCH_3_-benzene	14.8
**38**	3-OCH_3_-benzene	2.56
**39**	4-OCH_3_-benzene	6.27
**40**	3,4-diOCH_3_-benzene	13.8
**41**	2-OCF_3_-benzene	17.7
**42**	3-OCF_3_-benzene	1.42
**43**	4-OCF_3_-benzene	>20
**44**	2-CF_3_-benzene	1.45
**45**	3-CF_3_-benzene	1.22
**46**	4-CF_3_-benzene	>20
**47**	3,5-diCF_3_-benzene	>20
**48**	2-CN-benzene	9.17
**49**	3-CN-benzene	>20
**50**	4-CN-benzene	5.17
**51**	2-COOCH_3_-benzene	2.90
**52**	3-COOCH_3_-benzene	7.77
**53**	4-COOCH_3_-benzene	8.16
**54**	4-COOCH_2_CH_3_-benzene	19.4
**55**	4-COOH-benzene	0.75
**56**	2-SO_2_CH_3_	6.80
**57**	4-SO_2_CH_3_	>20
**58**	2-NO_2_-benzene	6.65
**59**	4-NO_2_-benzene	2.74
**60**	2-F,3-Cl-benzene	13.0
**61**	2-CH_3_-4-F-benzene	5.53
**62**	3-CF_3_-4-F-benzene	3.75
**63**	3-Cl, 4-CH_3_-benzene	8.14
**64**	2-Cl, 4-CF_3_-benzene	>20
**65**	3-CF_3_-5-Br-benzene	>20
**66**	2-pyridyl	1.07
**67**	3-pyridyl	15.1
**68**	2-thiophenyl	1.07
**69**	3-thiophenyl	12.5
**70**		3.05
**71**		7.10
**72**		19.1
**73**		>20
**74**		11.6

a Included as a comparator; see Ref. [32].

Clearly substitution on the benzene ring is required, with unsubstituted analogue **21** (IC_50_ = 8.46 μM) suffering a 7-fold loss in activity. While 2,4-difluoro-compound (**3**) was the original lead substitution pattern, it can be separated into the constituent mono-fluoro- analogues **22**–**24**. This reveals that the contribution of the 2- position is most important (IC_50_ = 2.03 μM), followed by 4- (9.65 μM), and lastly the 3-position which is detrimental to activity (>20 μM). Accordingly, the 3,4-difluoro-substituted compound **25** shows poor potency (18.6 μM) while that of the 2,4,6-trifluoro- analogue **26** is excellent (1.76 μM). This positional effect however, is not apparent in the corresponding chloro- compounds (**27**–**29**; IC_50_s 2.11–8.77 μM) where the SAR between 2-, 3- and 4- is relatively flat. This may help explain why the most potent compound of the set is the 3,4-dichloro- **30** (1.32 μM) and not the 2,4-dichloro- target **31** (15.0 μM) as might be expected from the fluorinated series of analogues. The bromo- (**32**–**35**; IC_50_s 2.11–8.77 μM) and iodo-substituted (**36**; 5.00 μM) compounds also displayed flat SAR, perhaps because all these halogens are less electron-withdrawing than fluorine, a concept that we explore in more detail below. A different trend was observed when the electron-donating substituents methoxy (**37**–**40**) and trifluoromethoxy (**41**–**43**) were employed; here the *meta*- position was favoured over *ortho*- and *para*-. The two *meta*-substituted examples **38** (2.56 μM) and **42** (1.42 μM) showed significantly better activity in comparison to other positional isomers. We then surveyed a variety of electron-withdrawing substituents by preparing compounds **44**–**59**. As a group, these broadly paralleled the fluorine-substituted compounds discussed above, where the *ortho*- or *para*- positional isomers showed superior potency over the corresponding *meta*- examples. More specifically, for the trifluoromethyl- (**44**–**47**), ester- (**51**–**54**) and sulphonyl- (**56**, **57**) substituted compounds, the *ortho* isomer was best (IC_50_s = 1.45, 2.90, 6.80 μM respectively), while for the cyano- (**48**–**50**), carboxylic acid (**55**) and nitro- (**58**, **59**) examples the *para* isomer showed high potency (5.17, 0.75 and 2.74 μM respectively). Compound **55** was particularly noteworthy, being one of the few sub-micromolar inhibitors of perforin identified to date. This subset of results is consistent with an inductive effect being exerted by electron-withdrawing substituents on the benzene ring and through to the sulphonamide, enhancing interactions with the protein and resulting in improved activity.

Hybrid compounds **60**–**65** were also prepared to investigate whether the effects of individual substituents could be combined. The resulting activities were neither synergistic nor additive, bringing no further gain to the overall potency. A set of four compounds (**66**–**69**) with a heterocycle (pyridine or thiophene) linked to the sulphonamide were also prepared. The preference for the heteroatom to be located directly next to the sulphonamide bond was clear with the 2-pyridyl and 2-thiophenyl compounds **66** and **68** (both IC_50_s = 1.07 μM) far superior to the corresponding 3-linked isomers **67** and **69** (15.13 and 12.51 μM respectively). Finally, a set of compounds containing a variety of substituted heterocycles were prepared (**70**–**74**), but with the exception of the 4-oxazole **70** (IC_50_ = 3.05 μM), none showed much promise.

### 2.3 Advanced assessment of selected compounds

Having shown that a range of benzenesulphonamides block lysis by recombinant perforin, a subset of promising examples was identified to test for inhibitory effect on the lytic action of whole NK cells. Compounds were selected based on potency, and included several for which the Jurkat IC_50_s were >20 μM, to further validate our use of this higher throughput screen as our primary assay. The inhibitors were co-incubated with KHYG1 human NK cells in medium for 30 min at room temperature, ^51^Cr-labelled target cells were added, and the resulting level of chromium release used to determine residual lytic activity and thus degree of inhibition. The use of whole NK cells to deliver perforin provides a more realistic model of conditions *in vivo* compared to isolated recombinant protein which acts indiscriminately. Recognition of a+ target cell, formation of a synaptic cleft, and release of the granular contents into the cavity between effector and target are all required elements for lysis to occur. Confirmation that the observed level of inhibition is due to blocking the activity of perforin rather than non-specific killing of the effector cell was also sought by measuring the viability of the NK cells 24 h later. Our lead compound for the current work and most potent compound from our previous study [32], 2,4-difluorobenzene **3**, is included as a reference point (Table 4). One notable omission from this table is the potent 4-carboxylic acid-substituted compound **55** as this was toxic to the NK cells and therefore the degree of inhibition was unable to be determined.

**Table 4:** Capacity of selected compounds to inhibit perforin delivered by KHYG1 NK cells.

Compound	Jurkat IC_50_ (μM)^a^	KHYG1 Inhibition (% at 10 μM)^b^	KHYG1 Viability (% at 10 μM)^c^
**3**	1.17	67.5 ± 17.5	100
**10**	1.99	92.5 ± 1.8	92.0 ± 3.0
**11**	1.03	95.0 ± 2.5	94.0 ± 5.3
**13**	>20	50.3 ± 8.5	91.7 ± 10.0
**16**	>20	0	96.1 ± 6.0
**17**	>20	0	91.4 ± 6.5
**26**	1.76	81.3 ± 6.3	95.1 ± 2.1
**44**	1.45	85.0 ± 5.6	100
**45**	1.22	88.8 ± 1.9	100
**47**	>20	0	90.2 ± 3.4
**49**	>20	83.7 ± 3.2	94.4 ± 7.1
**50**	5.17	75.0 ± 7.8	99.3 ± 0.3
**57**	>20	0	90.0 ± 7.8
**58**	6.65	95.6 ± 1.9	98.7 ± 1.5
**59**	2.74	77.5 ± 5.6	85.0 ± 12.0

a Data given for compounds as determined by the Jurkat assay.

b Inhibition by compound (10 μM) of the perforin-induced lysis of ^51^Cr-labelled K562 leukemia target cells when co-incubated with KHYG1 human NK cells. Percent inhibition calculated compared to untreated control (n = 4).

c Viability of KHYG1 NK cells after 24 h by Trypan blue exclusion assay (n = 3). See Experimental section for further details.

All of the compounds with Jurkat IC_50_s < 10 μM showed excellent suppression of NK-cell mediated killing of labelled target cells (68–96% at 10 μM), however this activity did not correlate exactly with their potency against isolated recombinant protein. This finding may reflect the varying ability of the respective inhibitors to access perforin located in a synaptic cleft within a complex biological milieu. Two examples with halogen on the central pyridine ring (**10**, **11**) and the 2-nitrobenzene compound **58** were particularly effective in blocking NK cell action (93, 95 and 96% inhibition respectively). Results from the set of compounds with IC_50_s > 20 μM broadly validated our use of this assay as a primary screen, with four of six examples showing no potency in either assay (**16**, **17**, **47**, **57**). Compound **13** demonstrated 50% inhibition at 10 μM, perhaps not surprisingly given that the only structural change from **3** was the replacement of a bridging pyridine with a benzene ring. The one unexpected outlier was the 3-cyano compound **49** which, although it had poor activity against isolated recombinant perforin, had excellent activity against perforin produced by whole NK cells. The NK cells also retained excellent viability across all examples, consistent with our previous findings for this series [32] and in contrast to earlier reported classes [26], [27], [28].

Preliminary physicochemical data was collected on the same (active) subset of compounds in order to assess their potential for progression to *in vivo* pharmacokinetic (PK) studies (Table 5). Following conversion to the corresponding sodium salts the solubility varied widely, with the 2,4,6-trifluorobenzene (**26**) and 4-cyanobenzene (**50**) analogues being highly soluble, while the presence of 2-fluoropyridine (**10**), 2-nitrobenzene or the more lipophilic trifluoromethylbenzene group (**44**, **45**) had a negative impact on solubility. All examples tested showed good stability in aqueous solution over 24 h, however results were more varied in the presence of human, rat and mouse microsomes. While **10**, **11**, and **58** showed acceptable stability (>70% parent after 30 min) across all three species, the remaining compounds (**3**, **26**, **45**, **50**, **59** and especially **44**) showed moderate to poor stability with human microsomes. This data in combination with poor solubility resulted in the elimination of **44** and **45** from consideration for the *in vivo* PK studies reported in section 2.4 below.

**Table 5:** Physicochemical properties of selected compounds.

Compound	Solubility^a^ (μg/mL)	cLogP^b^	Stability in Solution^c^ (%)	Microsome Stability (%)^d^		
				Mouse	Rat	Human
**3**^e^	1080	2.70 ± 0.72	82	92	99	58
**10**	428	2.34 ± 0.79	100	96	100	94
**11**	4027	3.21 ± 0.73	100	97	99	95
**26**	10674	2.65 ± 0.77	88	100	83	63
**44**	88	2.97 ± 0.65	92	84	49	14
**45**	574	3.13 ± 0.65	–	97	80	57
**50**	12900	2.17 ± 0.64	85	87	64	52
**58**	671	1.83 ± 0.61	100	100	89	73
**59**	1680	2.36 ± 0.61	–	85	72	39

a Solubility of the sodium salt in water at room temperature; conversion to salt as described in experimental section 4.1.

b cLogP calculated using ACD/PhysChem software v12.5.

c Percentage of parent compound (as sodium salt) remaining after 24 h at 20 °C in water.

d Percentage of parent compound (as sodium salt) remaining after exposure to mouse, rat or human microsomes for 30 min.

e Data for compound **3** from Ref. [32]. See Supplementary Material for further details of assay conditions.

### 2.4 *In vivo* pharmacokinetics

The *in vivo* PK parameters were measured for seven compounds selected on the basis of the *in vitro* assessment described above. Plasma pharmacokinetics were determined in male CD-1 mice for compounds **3**, **10**, **11**, **26**, **50**, **58** and **59** (Table 6). Blood samples were collected at 5–8 time-points after dosing the compounds at 10 mg/kg in a solution of 20% hydroxypropyl-β-cyclodextrin by intraperitoneal (IP) injection. For analysis of the samples, chromatographic conditions were optimised by HPLC for each compound of interest and an internal standard. A liquid chromatography with tandem mass spectrometry (LC-MS/MS) method was then developed and validated for quantitation of each analyte.

**Table 6:** *In vivo* Pharmacokinetics of selected compounds.^a^

Compound	T_1/2_ (h)	C_max_ (μmol/L)	AUC_0-∞_ (μmol/L*h)
**3**	12.0	10	220
**10**	6.6	236	2885
**11**	9.5	149	2364
**26**	4.5	124	1019
**50**	3.3	87	642
**58**	2.5	105	415
**59**	2.2	64	383

a Pharmacokinetic parameters derived from the plasma-concentration time profiles for each compound following a 10 mg/kg *i.p*. dose. The results were processed using a noncompartment model approach using Phoenix WinNonlin 6.2 (Pharsight Corporation, St. Louis, MO). The derived parameters are: maximum plasma concentration (C_max_), the area under the curve (AUC) and plasma half-life (T_1/2_). See Supplementary Material for further details of assay conditions.

Compound **3** possessed the best half-life (12 h) but showed much lower C_max_ and AUC than other examples. While **10**, **11** and **26** had shorter half-lives (4.5–9.5 h) they also showed the highest C_max_ (236, 149 and 124 μmol/L) and exposures (AUC = 2885, 2364 and 1019 μmol/L*h respectively). 4-Cyanobenzene (**50**) and both nitrobenzene isomers (**58**, **59**), had even shorter half-lives, however the maximum concentration and overall exposure reached was still superior to the compound with the overall longest half-life (**3**).

## 3 Conclusions

Between our previous report [32] and the current study, we have explored in detail how changes made on a benzenesulphonamide-based template affects perforin inhibitory activity. Analysis of the resulting SAR shows that although a range of variations were explored throughout the molecule, the isoindolinone, thiophene, pyridine and sulphonamide link of **3** are probably close to optimal, while there is some tolerance for substitution on the central pyridine ring and the terminal benzene ring. A smaller panel of compounds was selected based on the following criteria; *in vitro* potency against isolated recombinant perforin and whole human NK cells, lack of toxicity against NK cells, solubility and stability (aqueous and microsomal). For this group the *in vivo* PK parameters were assessed to select potential candidates for evaluation in a mouse model of transplant rejection. We sought acceptable half-life (potentially impacts dosing frequency), C_max_ and AUC to ensure sufficient exposure to maximise our chances of achieving efficacy. Other key parameters taken into consideration for selection of the final *in vivo* candidates were *in vitro* potency and solubility. While the *in vivo* mouse efficacy studies are carried out using IP dosing, ultimately this product would be administered to human patients intravenously, meaning that sufficient solubility in a formulation close to physiological pH is crucial.

Given the above criteria, one example from the panel that appears to possess a suitable balance of properties is the 2-chloro pyridine **11**. This compound shows excellent potency (without toxicity) *in vitro*, good solubility, stability across all three species of microsomes and acceptable PK properties. Compounds **58** and **3** are also worth consideration; **58** because it is the most potent compound of the set, and **3** based on a combination of potency, solubility and half-life. The next step will be to conduct studies to determine if these compounds are capable of preventing transplant rejection in a mouse model.

## 4 Experimental

### 4.1 Chemistry

Elemental analyses were performed by the Microchemical Laboratory, University of Otago, Dunedin, NZ; values are indicated by the symbols of the elements and were within ±0.4% of the theoretical values. Melting points were determined using an Electrothermal Model 9200 and are as read. Several compounds were examined as sharp-melting solvates, on which elemental analyses were determined. NMR spectra were measured on a Bruker Advance 400 MHz spectrometer and referenced to Me_4_Si. Mass spectra were recorded either on a Varian VG 7070 spectrometer at nominal 5000 resolution or a Finnigan MAT 900Q spectrometer. All final compound purities were determined to be >95% by HPLC on an Alltech Alltima C18 column (3.2 × 150 mm, 5 μm) eluting with 5–80% MeCN/45 mM NH_4_HCO_3_.

#### 4.1.1 General procedure A: 5-(5-(6-Aminopyridin-3-yl)thiophen-2-yl)-2-methylisoindolin-1-one (**76**)

Iodide **75** [32] (500 mg, 1.41 mmol) and 5-(4,4,5,5-tetramethyl-1,3,2-dioxaborolan-2-yl)pyridin-2-amine (465 mg, 2.11 mmol) were dissolved in a mixture of toluene (8 mL) and EtOH (4 mL). A solution of 2 M Na_2_CO_3_ (2 mL) and Pd(dppf)Cl_2_.CH_2_Cl_2_ (57 mg, 0.07 mmol) were added and the entire mixture heated at reflux under N_2_ for 2 h. Upon cooling, the desired product precipitated from the reaction mixture, was isolated by filtration, and washed with H_2_O, CH_2_Cl_2_ and MeOH. No further purification was required, giving **76** as a green-yellow solid (326 mg, 72%); mp (MeOH/CH_2_Cl_2_) 252–254 °C. ^1^H NMR [400 MHz, (CD_3_)_2_SO] δ 8.28 (dd, *J* = 2.6, 0.6 Hz, 1 H), 7.86 (s, 1 H), 7.76 (dd, *J* = 8.0, 1.5 Hz, 1 H), 7.70 (dd, *J* = 8.6, 2.6 Hz, 1 H), 7.67 (d, *J* = 7.9 Hz, 1 H), 7.62 (d, *J* = 3.8 Hz, 1 H), 6.35 (d, *J* = 3.8 Hz, 1 H), 6.51 (dd, *J* = 8.6, 0.6 Hz, 1 H), 6.25 (br s, 2 H) 4.49 (s, 2 H), 3.08 (s, 3 H). HRMS (ESI^+^) calcd for C_18_H_16_N_3_OS 322.1009 (MH^+^), found 322.1007.

#### 4.1.2 General procedure B: 2,4-Difluoro-*N*-(5-(5-(2-methyl-1-oxoisoindolin-5-yl)thiophen-2-yl)pyridin-2-yl)benzenesulfonamide (**4**)

To a solution of **76** (120 mg, 0.37 mmol) in dry pyridine (12 mL) under N_2_ at RT, was added 2,4-difluorobenzenesulphonyl chloride (159 mg, 0.74 mmol) in CH_2_Cl_2_ (1.5 mL) dropwise over 5 min. The mixture was stirred at 45 °C under N_2_ for 16 , and the solvent then removed under reduced pressure. The reaction was quenched with a little water and the resulting solid collected by filtration and washed with water and Et_2_O. Purification was carried out by trituration with hot CH_2_Cl_2_/MeOH solution to give **4** as a pale brown solid (65%); mp (CH_2_Cl_2_/MeOH) 269–272 °C. ^1^H NMR [400 MHz, (CD_3_)_2_SO] δ 12.00 (bs, 1 H), 8.39 (bs, 1 H), 7.99–8.10 (m, 2 H), 7.89 (s, 1 H), 7.78 (dd, *J* = 8.0, 1.3 Hz, 1 H), 7.69 (d, *J* = 4.2 Hz, 1 H), 7.68 (d, *J* = 8.1 Hz, 1 H), 7.57 (d, *J* = 3.9 Hz, 1 H), 7.44–7.53 (m, 1 H), 7.26–7.33 (m, 1 H), 7.16–7.26 (m, 1 H) 4.50 (s, 2 H), 3.07 (s, 3 H). HRMS (ESI^+^) calcd for C_24_H_18_N_3_O_3_S_2_F_2_ 498.0752 (MH^+^), found 498.0746. Anal. C, H, N. In some cases a bis-sulphonamide was also formed; here a second step was introduced where the crude product was treated with a 1:1 mixture of 1,4-dioxane and 2 M NaOH. The mono-sulphonamide resulting from subsequent acidification of the reaction mixture was isolated by filtration, washed well with water, and dried. Purification was carried out by flash column chromatography (MeOH/CH_2_Cl_2_ gradient).

#### 4.1.3 5-(5-(2-Aminopyridin-4-yl)thiophen-2-yl)-2-methylisoindolin-1-one (**77**)

Reaction of **75** with 4-(4,4,5,5-tetramethyl-1,3,2-dioxaborolan-2-yl)pyridin-2-amine according to general procedure A gave **77** as a green-yellow solid (72%); mp (CH_2_Cl_2_/MeOH) 302–306 °C. ^1^H NMR [400 MHz, (CD_3_)_2_SO] δ 7.90–7.97 (m, 2 H), 7.82 (d, *J* = 7.1 Hz, 1 H), 7.67–7.73 (m, 2 H), 7.65 (d, *J* = 3.4 Hz, 1 H), 6.83 (d, *J* = 4.0 Hz, 1 H), 6.69 (s, 1 H), 6.05 (br s, 2 H) 4.52 (s, 2 H), 3.09 (s, 3 H). HRMS (ESI^+^) calcd for C_18_H_16_N_3_OS 322.1009 (MH^+^), found 322.1002.

#### 4.1.4 2,4-Difluoro-*N*-(4-(5-(2-methyl-1-oxoisoindolin-5-yl)thiophen-2-yl)pyridin-2-yl)benzenesulfonamide (**5**)

Amine **77** was reacted with 2,4-difluorobenzenesulfonyl chloride according to general procedure B to give **5** as a pale brown solid (26%); mp (CH_2_Cl_2_/MeOH) 235–239 °C. ^1^H NMR [400 MHz, (CD_3_)_2_SO] δ 13.27 (br s, 1 H), 7.99–8.09 (m, 2 H), 7.85–7.98 (m, 3 H), 7.80 (d, *J* = 3.8 Hz, 1 H), 7.72 (d, *J* = 7.9 Hz, 1 H), 7.38–7.51 (m, 2 H), 7.19–7.32 (m, 2 H) 4.53 (s, 2 H), 3.09 (s, 3 H). HRMS (ESI^+^) calcd for C_24_H_18_N_3_O_3_S_2_F_2_ 498.0752 (MH^+^), found 498.0754. Anal. C, H, N.

#### 4.1.5 *N*-(5-Bromopyridin-3-yl)-2,4-difluoro-*N*-methylbenzenesulphonamide (**86**)

2,4-Difluorobenzenesulfonyl chloride and 5-bromopyridin-3-amine were reacted according to general procedure B. Without further purification, the resulting crude sulphonamide **85** (540 mg, 15.5 mmol) was dissolved in dry DMF (20 mL) and cooled to 0 °C. NaH (41 mg, 17.0 mmol) was added and the mixture stirred for 0.5 , gradually being allowed to return to R.T. Methyl iodide (241 mg, 17.0 mmol) was then added dropwise and stirring continued for 1.5 h. After quenching with water the mixture was extracted with CH_2_Cl_2_, dried with MgSO_4_ and evaporated to give a solid which was purified by flash column chromatography (1–3% MeOH/CH_2_Cl_2_ as eluant), yielding **86** as a brown solid (320 mg, 57%). ^1^H NMR [400 MHz, (CD_3_)_2_SO] δ 8.65 (d, *J* = 2.0 Hz, 1 H), 8.51 (d, *J* = 2.2 Hz, 1 H), 8.04 (t, *J* = 2.2 Hz, 1 H), 7.72–7.83 (m, 1 H), 7.57–7.68 (m, 1 H), 7.32 (dt, *J* = 8.2, 2.4 Hz, 1 H), 3.26 (s, 3 H). LRMS (APCI^+^) calcd for C_12_H_9_BrF_2_N_2_O_2_S 364 (MH^+^), found 364.

#### 4.1.6 General procedure C: 2,4-Difluoro-*N*-methyl-*N*-(5-(5-(2-methyl-1-oxoisoindolin-5-yl)thiophen-2-yl)pyridin-3-yl)-benzenesulphonamide (**6**)

Bromide **86** (300 mg, 0.83 mmol), bis(pinacolato)diboron (232 mg, 0.91 mmol), KOAc (243 mg, 2.48 mmol) and Pd(dppf)Cl_2_.CH_2_Cl_2_ (34 mg, 0.04 mmol) were weighed into a flask, DMSO (5 mL) added, and the entire mixture heated and stirred under N_2_ for 4 h. Upon cooling, the reaction was diluted with CH_2_Cl_2_ (25 mL) and filtered through a pad of Celite^®^, washing well with additional CH_2_Cl_2_. The filtrate and combined washings (ca 80 mL) were washed with water (3 × 40 mL), brine (50 mL), dried (Na_2_SO_4_) and filtered. Removal of the solvent under reduced pressure gave the crude boronate **90** which was coupled directly to **75** according to general procedure A, giving **6** as a cream solid (220 mg, 52%); mp (CH_2_Cl_2_/MeOH) 200–202 °C. ^1^H NMR [400 MHz, (CD_3_)_2_SO] δ 8.87 (d, *J* = 2.0 Hz, 1 H), 8.43 (d, *J* = 2.3 Hz, 1 H), 7.96 (t, *J* = 2.2 Hz, 1 H), 7.94 (s, 1 H), 7.83 (dd, *J* = 7.8, 1.4 Hz, 1 H), 7.73–7.81 (m, 3 H), 7.71 (d, *J* = 7.9 Hz, 1 H), 7.63 (m, 1 H), 7.32 (dt, *J* = 8.1, 2.0 Hz, 1 H), 4.52 (s, 2 H), 3.34 (s, 3 H), 3.09 (s, 3 H). Anal. C, H, N.

#### 4.1.7 5-Bromo-*N*-(2,4-difluorophenyl)pyridine-3-sulphonamide (**100**)

2,4-Difluoroaniline and 5-bromopyridine-3-sulphonyl chloride were reacted according to general procedure B. The crude product was recrystallised from 5% MeOH/CH_2_Cl_2_ and hexanes, and triturated with EtOAc to give **100** as an ivory solid (370 mg, 56%). ^1^H NMR [400 MHz, (CD_3_)_2_SO] δ 10.52 (br s, 1 H), 9.20 (d, *J* = 2.2 Hz, 1 H), 8.77 (d, *J* = 2.0 Hz, 1 H), 8.26 (t, *J* = 2.1 Hz, 1 H), 7.22–7.35 (m, 2 H), 7.02–7.15 (m, 1 H). LRMS (APCI^+^) calcd for C_11_H_7_BrF_2_N_2_O_2_S 350 (MH^+^), found 350.

#### 4.1.8 *N*-(2,4-Difluorophenyl)-5-(5-(2-methyl-1-oxoisoindolin-5-yl)thiophen-2-yl)pyridine-3-sulphonamide (**7**)

Bromide **100** was reacted with bis(pinacolato)diboron according to general procedure C and the crude boronate **101** subsequently coupled to **75** according to general procedure A, to give **7** as a yellow solid (23%); mp (CH_2_Cl_2_/MeOH) 221–224 °C. ^1^H NMR [400 MHz, (CD_3_)_2_SO] δ 10.50 (br s, 1 H), 9.23 (d, *J* = 2.2 Hz, 1 H), 8.71 (d, *J* = 2.1 Hz, 1 H), 8.21 (t, *J* = 2.2 Hz, 1 H), 7.96 (s, 1 H), 7.81–7.88 (m, 2 H), 7.78 (d, *J* = 3.9 Hz, 1 H), 7.72 (d, *J* = 8.0 Hz, 1 H), 7.23–7.35 (m, 2 H), 7.09 (ddt, *J* = 9.2, 1.4 Hz, 1 H), 4.52 (s, 2 H), 3.09 (s, 3 H). Anal. C, H, N.

#### 4.1.9 2,4-Difluoro-*N*-(5-(5-(2-methyl-1-oxoisoindolin-5-yl)thiophen-2-yl)pyridin-3-yl)benzamide (**8**)

To 2,4-difluorobenzoic acid (99 mg, 0.62 mmol) in dry CH_2_Cl_2_ (2 mL) was added oxalyl chloride (193 mg, 1.52 mmol) and 1 drop of dry DMF. The whole mixture was refluxed for 2 , cooled to RT and concentrated *in vacuo* to give 2,4-difluorobenzoic acid chloride. To amine **78** [32] (100 mg, 0.31 mmol) in dry pyridine (10 mL) at 0 °C under N_2_ was added the acid chloride (110 mg, 0.62 mmol) in dry CH_2_Cl_2_ (2 mL) dropwise over 4 min. The mixture was then left to stir at 45 °C for 16 , quenched with H_2_O and concentrated *in vacuo*. The residue was taken up in citric acid, sonicated for 5 min, and the precipitate formed was filtered and washed thoroughly with H_2_O, MeOH, diethyl ether and dried on to silica gel. The crude material was chromatographed (1–3% MeOH/CH_2_Cl_2_) to give **8** as a yellow solid (52 mg, 36%); mp 267–269 °C. ^1^H NMR [400 MHz, (CD_3_)_2_SO] δ 10.76 (br s, 1 H), 8.77 (t, *J* = 2.9 Hz, 2 H), 8.52 (t, *J* = 2.0 Hz, 1 H), 7.96 (s, 1 H), 7.81–7.88 (m, 2 H), 7.75 (d, *J* = 3.9 Hz, 1 H), 7.71 (d, *J* = 3.8 Hz, 1 H), 7.70 (s, 1 H), 7.49 (dt, *J* = 9.4, 2.5 Hz, 1 H), 7.28 (dt, *J* = 8.6, 2.2 Hz, 1 H) 4.52 (s, 2 H), 3.09 (s, 3 H). LRMS (APCI^+^) calcd for C_25_H_17_N_3_O_2_F_2_S 462.5 (MH^+^), found 462.8. Anal. C, H, N.

#### 4.1.10 5-(5-(3-Aminophenyl)thiophen-2-yl)-2-methylisoindolin-1-one (**79**)

Iodide **75** was reacted with (3-aminophenyl)boronic acid according to general procedure A to give **79** as a green solid (69%); mp (CH_2_Cl_2_/MeOH) 244–247 °C. ^1^H NMR [400 MHz, (CD_3_)_2_SO] δ 7.89 (s, 1 H), 7.77 (dd, *J* = 7.9, 1.3 Hz, 1 H), 7.68 (d, *J* = 8.0 Hz, 1 H), 7.63 (d, *J* = 3.8 Hz, 1 H), 7.41 (d, *J* = 3.8 Hz, 1 H), 7.08 (t, *J* = 7.72 Hz, 1 H), 6.82–6.91 (m, 2 H), 6.55 (dd, *J* = 7.9, 1.3 Hz, 1 H), 5.25 (br s, 2 H), 4.50 (s, 2 H), 3.08 (s, 3 H).

#### 4.1.11 2,4-Difluoro-*N*-(3-(5-(2-methyl-1-oxoisoindolin-5-yl)thiophen-2-yl)phenyl)benzene sulphonamide (**9**)

Amine **79** was reacted with 2,4-difluorobenzenesulphonyl chloride according to general procedure B to give **9** as a yellow solid (62%); mp (CH_2_Cl_2_/MeOH) 260–262 °C. ^1^H NMR [400 MHz, (CD_3_)_2_SO] δ 10.84 (br s, 1 H), 7.93–8.02 (m, 1 H), 7.91 (s, 1 H), 7.81 (dd, *J* = 8.0, 1.3 Hz, 1 H), 7.70 (d, *J* = 8.0 Hz, 1 H), 7.67 (d, *J* = 3.8 Hz, 1 H), 7.55 (dt, *J* = 8.5, 2.5 Hz, 1 H), 7.47 (d, *J* = 3.8 Hz, 1 H), 7.38–7.45 (m, 2 H), 7.25–7.36 (m, 2 H), 7.05 (d, *J* = 7.4 Hz, 1 H), 4.51 (s, 2 H), 3.09 (s, 3 H). Anal. C, H, N.

#### 4.1.12 *N*-(5-Bromo-2-fluoropyridin-3-yl)-2,4-difluorobenzenesulphonamide (**82**)

5-Bromo-2-fluoropyridin-3-amine was reacted with 2,4-difluorobenzenesulphonyl chloride according to general procedure B to give **82** as a brown solid (17%). ^1^H NMR [400 MHz, (CD_3_)_2_SO] δ 11.13 (br s, 1 H), 8.18 (s, 1 H), 8.01 (dd, *J* = 8.6, 2.3 Hz, 1 H), 7.81–7.92 (m, 1 H), 7.53–7.64 (m, 1 H), 7.22–7.32 (m, 1 H). LRMS (APCI^+^) calcd for C_11_H_6_BrF_3_N_2_O_2_S 368 (MH^+^), found 368.

#### 4.1.13 2,4-Difluoro-*N*-(2-fluoro-5-(5-(2-methyl-1-oxoisoindolin-5-yl)thiophen-2-yl)pyridin-3-yl)benzenesulphonamide (**10**)

Bromide **82** was reacted with bis(pinacolato)diboron according to general procedure C and the crude boronate **87** subsequently coupled to **75** according to general procedure A to give **10** as a pale green solid (32%); mp (MeOH/CH_2_Cl_2_) 237–239 °C. ^1^H NMR [400 MHz, (CD_3_)_2_SO] δ 11.04 (br s, 1 H), 8.42 (s, 1 H), 8.06 (dd, *J* = 9.1, 2.3 Hz, 1 H), 7.94 (s, 1 H), 7.84–7.92 (m, 1 H), 7.82 (dd, *J* = 7.9, 1.5 Hz, 1 H), 7.74 (d, *J* = 3.9 Hz, 1 H), 7.72 (d, *J* = 8.0 Hz, 1 H), 7.68 (d, *J* = 3.9 Hz, 1 H), 7.57–7.65 (m, 1 H), 7.28 (dt, *J* = 8.9, 2.4 Hz, 1 H), 4.52 (s, 2 H), 3.09 (s, 3 H). In this case the product was dissolved in 1,4-dioxane and the sodium salt precipitated by slow addition of 2 M NaOH to give a light-green solid (89%). ^1^H NMR [400 MHz, (CD_3_)_2_SO] δ 7.91 (s, 1 H), 7.83–7.90 (m, 1 H), 7.79 (dd, *J* = 7.9, 1.5 Hz, 1 H), 7.71 (d, *J* = 2.3 Hz, 1 H), 7.67 (d, *J* = 7.3 Hz, 1 H), 7.60–7.65 (m, 2 H), 7.33 (d, *J* = 3.8 Hz, 1 H), 7.22 (dt, *J* = 9.7, 2.5 Hz, 1 H), 7.11 (dt, *J* = 8.3, 2.5 Hz, 1 H), 4.51 (s, 2 H), 3.08 (s, 3 H). Anal. C, H, N.

#### 4.1.14 *N*-(5-Bromo-2-chloropyridin-3-yl)-2,4-difluorobenzenesulphonamide (**83**)

5-Bromo-2-chloropyridin-3-amine was reacted with 2,4-difluorobenzenesulphonyl chloride according to general procedure B, giving **83** as an off-white solid (32%). ^1^H NMR [400 MHz, (CD_3_)_2_SO] δ 11.03 (br s, 1 H), 8.47 (d, *J* = 2.3 Hz, 1 H), 8.04 (d, *J* = 2.3 Hz, 1 H), 7.75–7.85 (m, 1 H), 7.53–7.63 (m, 1 H), 7.20–7.29 (m, 1 H). LRMS (APCI^+^) calcd for C_11_H_6_BrF_2_ClN_2_O_2_S 385 (MH^+^), found 385.

#### 4.1.15 2,4-Difluoro-*N*-(2-chloro-5-(5-(2-methyl-1-oxoisoindolin-5-yl)thiophen-2-yl)pyridin-3-yl)benzenesulphonamide (**11**)

Bromide **83** was reacted with bis(pinacolato)diboron according to general procedure C and the crude boronate **88** subsequently coupled to **75** according to general procedure A to give **11** as a yellow solid (12%); mp (CH_2_Cl_2_/MeOH) 238–240 °C. ^1^H NMR [400 MHz, (CD_3_)_2_SO] δ 10.92 (br s, 1 H), 8.68 (d, *J* = 1.8 Hz, 1 H), 8.02 (d, *J* = 2.4 Hz, 1 H), 7.96 (s, 1 H), 7.84 (dd, *J* = 8.2, 1.8 Hz, 1 H), 7.74–7.85 (m, 3 H), 7.71 (d, *J* = 8.0 Hz, 1 H), 7.55–7.65 (m, 1 H), 7.26 (dt, *J* = 8.5, 2.0 Hz, 1 H), 4.52 (s, 2 H), 3.09 (s, 3 H). Anal. C, H, N.

#### 4.1.16 *N*-(5-Bromo-2-methoxypyridin-3-yl)-2,4-difluorobenzenesulphonamide (**84**)

5-Bromo-2-methoxypyridin-3-amine was reacted with 2,4-difluorobenzenesulphonyl chloride according to general procedure B, giving **84** as an ivory solid (45%). ^1^H NMR [400 MHz, (CD_3_)_2_SO] δ 10.44 (br s, 1 H), 8.12 (d, *J* = 2.3 Hz, 1 H), 7.72–7.81 (m, 1 H), 7.75 (d, *J* = 2.3 Hz, 1 H), 7.52–7.61 (m, 1 H), 7.18–7.27 (m, 1 H), 3.61 (s, 3 H). LRMS (APCI^+^) calcd for C_12_H_9_BrF_2_N_2_O_3_S 380 (MH^+^), found 380.

#### 4.1.17 2,4-Difluoro-*N*-(2-methoxy-5-(5-(2-methyl-1-oxoisoindolin-5-yl)-thiophen-2-yl)pyridin-3-yl)benzenesulphonamide (**12**)

Bromide **84** was reacted with bis(pinacolato)diboron and the crude boronate **89** subsequently coupled to **75** according to general procedure A, to give **12** as a yellow powder (42%); mp (MeOH/CH_2_Cl_2_) 224–227 °C. ^1^H NMR [400 MHz, (CD_3_)_2_SO] δ 10.35 (br s, 1 H), 8.35 (s, 1 H), 7.92 (s, 1 H), 7.85 (d, *J* = 2.2 Hz, 1 H), 7.82 (dd, *J* = 8.0, 1.5 Hz, 1 H), 7.74–7.83 (m, 1 H), 7.70 (d, *J* = 7.3 Hz, 1 H), 7.69 (d, *J* = 4.0 Hz, 1 H), 7.51–7.61 (m, 1 H), 7.54 (d, *J* = 3.7 Hz, 1 H), 7.22 (dt, *J* = 8.3, 2.3 Hz, 1 H), 4.51 (s, 2 H), 3.66 (s, 3 H), 3.09 (s, 3 H). Anal. C, H, N.

#### 4.1.18 2-Methyl-5-(5-(2-methyl-5-nitrophenyl)thiophen-2-yl)isoindolin-1-one (**80**)

Iodide **75** was reacted with (2-methyl-5-nitrophenyl)boronic acid according to general procedure A, followed by flash column chromatography (CH_2_Cl_2_/MeOH 98:2 as eluant) to give **80** as a yellow solid (80%). ^1^H NMR [400 MHz, CDCl_3_] δ 8.32 (d, *J* = 2.5 Hz, 1 H), 8.11 (dd, *J* = 8.4, 2.5 Hz, 1 H), 7.87 (d, *J* = 7.9 Hz, 1 H), 7.74 (dd, *J* = 7.9, 1.5 Hz, 1 H), 7.69 (dd, *J* = 1.4, 0.7 Hz, 1 H), 7.46 (d, *J* = 8.4 Hz, 1 H), 7.42 (d, *J* = 3.8 Hz, 1 H), 7.16 (d, *J* = 3.8 Hz, 1 H), 4.43 (s, 2 H), 3.23 (s, 3 H), 2.59 (s, 3 H). LRMS (APCI^+^) calcd for C_20_H_17_N_2_O_3_S 365 (MH^+^), found 365.

#### 4.1.19 2,4-Difluoro-*N*-(4-methyl-3-(5-(2-methyl-1-oxoisoindolin-5-yl)thiophen-2-yl)phenyl)benzenesulfonamide (**13**)

A 200 mL Parr hydrogenation vessel was charged with **80** (388 mg, 1.06 mmol) which was dissolved in a 1:1:1 mixture of ethanol, EtOAc and THF (90 mL). The mixture was agitated under 60 psi hydrogen at RT for 3 , before being filtered through Celite^®^. The solvents were evaporated to give the crude aniline **81** (350 mg) of which a subsample (111 mg, 0.33 mmol) was reacted with 2,4-difluorobenzenesulfonyl chloride according to general procedure B, giving **13** as a cream solid (51 mg, 30%); mp (MeOH/CH_2_Cl_2_) 268–271 °C. ^1^H NMR [400 MHz, (CD_3_)_2_SO] δ 10.68 (s, 1 H), 7.88–7.95 (m, 2 H), 7.80 (dd, *J* = 8.0, 1.5 Hz, 1 H), 7.70 (d, *J* = 8.0 Hz, 1 H), 7.67 (d, *J* = 3.8 Hz, 1 H), 7.56 (ddd, *J* = 11.4, 9.2, 2.4 Hz, 1 H), 7.29 (dt, *J* = 8.5, 8.4, 2.1 Hz, 1 H), 7.18–7.24 (m, 2 H), 7.03 (dd, *J* = 8.2, 2.4 Hz, 1 H), 4.52 (s, 2 H), 3.09 (s, 3H), 2.33 (s, 3 H). Anal. C, H, N.

#### 4.1.20 General procedure D: 3-Bromo-*N*-(ethoxymethyl)-*N*-(pyridin-2-yl)benzenesulfonamide (**105**)

Reaction of pyridine-2-amine and 3-bromobenzenesulfonyl chloride was carried out according to general procedure B. Without any further purification, to a solution of the crude sulphonamide **103** (500 mg, 1.60 mmol) and (chloromethoxy)ethane (166 mg, 1.76 mmol) in DMF at RT was added NaH (42 mg, 1.76 mmol), then the reaction stirred for 1 h. After quenching with water the mixture was extracted with CH_2_Cl_2_, dried with MgSO_4_ and evaporated to give a solid which was purified by flash chromatography on silica gel (3:1 hexanes/EtOAc) giving **105** as a colourless oil (373 mg, 63%). ^1^H NMR [400 MHz, CDCl_3_] δ 8.35 (ddd, *J* = 4.8, 1.9, 0.8 Hz, 1 H), 7.98 (dd, *J* = 1.8, 1.8 Hz, 1 H), 7.69–7.74 (m, 3 H), 7.66 (ddd, *J* = 8.1, 1.9, 1.0 Hz, 1 H), 7.48 (ddd, *J* = 8.2, 0.8, 0.8 Hz, 1 H), 7.32 (dd, *J* = 8.0, 8.0 Hz, 1 H), 7.16 (ddd, *J* = 7.4, 4.9, 1.0 Hz, 1 H), 5.38 (s, 2 H), 3.68 (q, *J* = 7.1 Hz, 2 H), 1.19 (t, *J* = 7.1 Hz, 3 H). LRMS (APCI^+^) calcd for C_12_H_10_BrFN_2_O_2_S 325 (M-EtO)^+^, found 325.

#### 4.1.21 General procedure E: sodium ((3-(5-(2-methyl-1-oxoisoindolin-5-yl)thiophen-2-yl)phenyl)sulfonyl)(pyridin-2-yl)amide (**14**)

Bromide **105** was reacted with bis(pinacolato)diboron and the crude boronate **108** subsequently coupled to **75** according to general procedure A. After extraction of the reaction mixture with EtOAc and evaporation, the protected crude intermediate was subjected to a one-pot deprotection and conversion to the sodium salt as follows; the solid was taken up in a 1:1 solution of 3 M HCl and 1,4-dioxane and then heated to reflux for 1 h. Upon cooling the white precipitate, consisting of essentially pure sulfonamide, was filtered and taken up in EtOH. Precipitation of the sodium salt was accomplished by slow addition of 2 M NaOH to give **14** as a yellow solid (68%, over 3 steps); mp (MeOH/CH_2_Cl_2_) 302–306 °C. ^1^H NMR [400 MHz, (CD_3_)_2_SO] δ 8.09 (dd, *J* = 1.6, 1.6 Hz, 1 H), 7.94 (br s, 1 H), 7.87 (ddd, *J* = 4.9, 2.1, 0.7 Hz, 1 H), 7.83 (dd, *J* = 8.0, 1.5 Hz, 1 H), 7.68–7.72 (m, 4 H), 7.56 (d, *J* = 3.9 Hz, 1 H), 7.41 (dd, *J* = 7.8, 7.8 Hz, 1 H), 7.19 (ddd, *J* = 8.5, 7.0, 2.2 Hz, 1 H), 6.59 (ddd, *J* = 8.6, 0.9, 0.9 Hz, 1 H), 6.36 (ddd, *J* = 7.0, 5.0, 1.0 Hz, 1 H), 4.51 (s, 2 H), 3.08 (s, 3 H). Anal. C, H, N.

#### 4.1.22 *N*-(4-Methyl-3-(5-(2-methyl-1-oxoisoindolin-5-yl)thiophen-2-yl)phenyl)pyridine-2-sulfonamide (**15**)

Amine **81** was reacted with pyridine-2-sulfonyl chloride according to general procedure B to give **15** as a cream solid (66 mg, 42%); mp (MeOH/CH_2_Cl_2_) 274–277 °C. ^1^H NMR [400 MHz, (CD_3_)_2_SO] δ 10.58 (s, 1 H), 8.74 (ddd, *J* = 4.65, 1.6, 0.8 Hz, 1 H), 8.08 (dt, *J* = 7.7, 7.7, 1.7 Hz, 1 H), 7.98 (td, *J* = 7.9, 1.0, 1.0 Hz, 1 H), 7.90 (s, 1 H), 7.80 (dd, *J* = 7.9, 1.6 Hz, 1 H), 7.70 (d, *J* = 8.0 Hz, 1 H), 7.64–7.68 (m, 2 H), 7.26 (d, *J* = 2.3 Hz, 1 H), 7.16–7.20 (m, 2 H), 7.06 (dd, *J* = 8.2, 2.3 Hz, 1 H), 4.51 (s, 2 H), 3.09 (s, 3 H), 2.32 (s, 3 H). Anal. C, H, N.

#### 4.1.23 Sodium (4-methyl-3-(5-(2-methyl-1-oxoisoindolin-5-yl)thiophen-2-yl)phenylsulfonyl)(pyridin-2-yl)amide (**16**)

3-Bromo-4-methylbenzenesulfonyl chloride and 2-aminopyridine were reacted according to general procedure B to give **102**. This was then reacted with bis(pinacolato)diboron according to general procedure C and the crude boronate **107** was coupled to **75** according to general procedure A to give **16**. Conversion to the sodium salt using general procedure E afforded a yellow solid (55%, 4 steps). ^1^H NMR [400 MHz, (CD_3_)_2_SO] δ 7.93 (br s, 1 H), 7.87–7.88 (m, 2 H), 7.83 (dd, *J* = 8.0, 1.4 Hz, 1 H), 7.68–7.69 (m, 2 H), 7.64 (dd, *J* = 7.9, 1.8 Hz, 1 H), 7.30 (d, *J* = 8.0 Hz, 1 H), 7.25 (d, *J* = 3.8 Hz, 1 H), 7.18 (ddd, *J* = 8.6, 7.0, 2.2 Hz, 1 H), 6.58 (ddd, *J* = 8.6, 1.0, 1.0 Hz, 1 H), 6.35 (ddd, *J* = 7.0, 5.0, 1.0 Hz, 1 H), 4.51 (s, 2 H), 3.08 (s, 3 H), 2.44 (s, 3 H). Anal. C, H, N.

#### 4.1.24 *N*-{4-Fluoro-3-[5-(2-methyl-1-oxo-2,3-dihydro-1*H*-isoindol-5-yl)-2-thienyl]phenyl}-2-pyridinesulfonamide (**17**)

2-Pyridinesulfonyl chloride and 3-bromo-4-fluoroaniline were reacted according to general procedure B to give **91**. Protection of the sulfonamide nitrogen was then carried out according to general procedure D, giving **94** which was purified by column chromatography eluting with hexanes/EtOAc 3:1. The protected sulphonamide was then reacted directly with bis(pinacolato)diboron according to general procedure C and the crude boronate **97** coupled to **75** according to general procedure A. Deprotection according to general procedure E gave **17** (11%, 5 steps) ^1^H NMR [400 MHz, (CD_3_)_2_SO] δ 8.13 (br s, 1 H), 8.01 (br s, 1 H), 7.88–7.97 (m, 3 H), 7.83 (dd, *J* = 8.0, 1.6 Hz, 1 H), 7.79–7.81 (m, 1 H), 7.75–7.77 (m, 1 H), 7.68–7.73 (m, 3 H), 7.61 (t, *J* = 7.8 Hz, 1 H), 7.24 (d, *J* = 8.4 Hz, 1 H), 6.87 (t, *J* = 6.9 Hz, 1 H), 4.51 (s, 2 H), 3.08 (s, 3 H). Anal. C, H, N.

#### 4.1.25 *N*-(5-Bromo-2-(trifluoromethoxy)phenyl)-*N*-(ethoxymethyl)pyridine-2-sulfonamide (**95**)

Pyridine-2-sulfonyl chloride and 5-bromo-2-(trifluoromethoxy)aniline were reacted according to general procedure B to give **92**. Protection of the sulfonamide according to general procedure D, gave **95** which was purified by column chromatography eluting with hexanes/EtOAc 3:1 and isolated as a colourless oil (89%, 2 steps).^1^H NMR [400 MHz, CDCl_3_] δ 8.75 (ddd, *J* = 4.8, 1.6, 1.0 Hz, 1H), 7.82–7.90 (m, 2 H), 7.46–7.54 (m, 3 H), 7.10 (dddd, *J* = 8.9, 1.9, 1.8, 1.8 Hz, 1 H), 5.21 (br s, 2 H), 3.79 (q, *J* = 7.0 Hz, 2 H), 1.20 (t, *J* = 7.0 Hz, 3 H). LRMS (APCI^+^) calcd for C_13_H_10_BrF_3_N_2_O_3_S 409 (M-EtO)^+^, found 409.

#### 4.1.26 *N*-(5-(5-(2-Methyl-1-oxoisoindolin-5-yl)thiophen-2-yl)-2-(trifluoromethoxy)phenyl)pyridine-2-sulfonamide (**18**)

Bromide **95** was reacted with bis(pinacolato)diboron, to give the crude boronate **98** which was subsequently coupled to **75** according to general procedure A. The intermediate from this step was then deprotected according to general procedure E giving **18** as a yellow solid (51%, 3 steps); mp (MeOH/CH_2_Cl_2_) 200–201 °C. ^1^H NMR [400 MHz, (CD_3_)_2_SO] δ 10.61 (s, 1 H), 8.79 (ddd, *J* = 4.6, 1.6, 0.8 Hz, 1 H), 8.11 (ddd, *J* = 7.7, 7.7, 1.7 Hz, 1 H), 7.99 (ddd, *J* = 7.9, 0.9, 0.9 Hz, 1 H), 7.93–7.95 (m, 1 H), 7.82 (dd, *J* = 8.0, 1.5 Hz, 1 H), 7.79 (d, *J* = 2.3 Hz, 1 H), 7.68–7.74 (m, 3 H), 7.61 (dd, *J* = 8.6, 2.3 Hz, 1 H), 7.53 (d, *J* = 3.4 Hz, 1 H), 7.39 (dddd, *J* = 8.5, 1.5, 1.5, 1.5 Hz, 1 H), 4.52 (s, 2 H), 3.09 (s, 3 H). Anal. C, H, N.

#### 4.1.27 5-Bromo-*N*-(ethoxymethyl)-2-methoxy-*N*-(pyridin-2-yl)benzenesulfonamide (**106**)

Pyridine-2-amine and 5-bromo-2-methoxybenzenesulfonyl chloride were reacted according to general procedure B. Protection of the sulfonamide **104** was then carried out according to general procedure D, giving **106** as a colourless oil (99%, 2 steps). ^1^H NMR [400 MHz, CDCl_3_] δ 8.76 (ddd, *J* = 4.7, 1.6, 0.8 Hz, 1 H), 7.82 (dd, *J* = 7.7, 1.7 Hz, 1 H), 7.76 (ddd, *J* = 7.9, 1.1, 1.1 Hz, 1 H), 7.48 (ddd, *J* = 7.6, 4.8, 1.3 Hz, 1 H), 7.37–7.40 (m, 2 H), 6.64–6.68 (m, 1 H), 5.19 (br s, 2 H), 3.80 (q, *J* = 7.0 Hz, 2 H), 3.37 (s, 3 H), 1.21 (t, *J* = 7.0 Hz, 3 H). LRMS (APCI^+^) calcd for C_13_H_13_BrN_2_O_3_S 355 (M-EtO)^+^, found 355.

#### 4.1.28 Sodium ((2-methoxy-5-(5-(2-methyl-1-oxoisoindolin-5-yl)thiophen-2-yl)phenyl)sulfonyl)(pyridin-2-yl)amide (**19**)

Bromide **106** was reacted with bis(pinacolato)diboron according to general procedure C, then the crude boronate **109** was subsequently coupled to **75** according to general procedure A. The intermediate from this step was deprotected and converted to the sodium salt according to general procedure E, furnishing **19** as a yellow solid (68%, 3 steps); mp (MeOH/CH_2_Cl_2_) 322–325 °C. ^1^H NMR [400 MHz, (CD_3_)_2_SO] δ 8.10 (br d, *J* = 2.5 Hz, 1 H), 7.89 (br s, 1 H), 7.86 (ddd, *J* = 4.9, 2.1, 0.8 Hz, 1 H), 7.79 (dd, *J* = 8.0, 1.5 Hz, 1 H), 7.63–7.68 (m, 3 H), 7.36 (d, *J* = 3.8 Hz, 1 H), 7.20 (ddd, *J* = 8.4, 7.0, 2.1 Hz, 1 H), 7.04 (d, *J* = 8.7 Hz, 1 H), 6.71 (br d, *J* = 8.4 Hz, 1 H), 6.36 (ddd, *J* = 6.9, 4.8, 1.0 Hz, 1 H), 4.50 (s, 2 H), 3.74 (s, 3 H), 3.08 (s, 3 H). Anal. C, H, N.

#### 4.1.29 *N*-(5-Bromo-2-fluoropyridin-3-yl)-*N*-(ethoxymethyl)pyridine-2-sulfonamide (**96**)

Pyridine-2-sulfonyl chloride and 5-bromo-2-fluoropyridin-3-amine were reacted according to general procedure B. Intermediate **93** was then protected according to general procedure D to give **96** as a colourless oil (32%, 2 steps). ^1^H NMR [400 MHz, (CD_3_)_2_SO] δ 8.75 (ddd, *J* = 4.7, 1.6, 0.9 Hz, 1 H), 8.22 (dd, *J* = 2.4, 1.5 Hz, 1 H), 8.01 (dd, *J* = 8.1, 2.4 Hz, 1 H), 7.84–7.92 (m, 2 H), 7.54 (ddd, *J* = 7.4, 4.7, 1.4 Hz, 1 H), 5.21 (s, 2 H), 3.76 (q, *J* = 7.1 Hz, 2 H), 1.20 (t, *J* = 7.0 Hz, 3 H). LRMS (APCI^+^) calcd for C_11_H_8_BrFN_3_O_2_S 346 (M-EtO)^+^, found 346.

#### 4.1.30 *N*-(2-Fluoro-5-(5-(2-methyl-1-oxoisoindolin-5-yl)thiophen-2-yl)pyridin-3-yl)pyridine-2-sulfonamide (**20**)

Bromide **96** was reacted with bis(pinacolato)diboron to afford crude boronate **99** which was subsequently coupled to **75** according to general procedure A. The intermediate from this step was then deprotected according to general procedure E to give **20** as a white solid (11%, 3 steps); mp (CH_2_Cl_2_/MeOH) 279–382 °C. ^1^H NMR [400 MHz, (CD_3_)_2_SO] δ 10.9 (br s, 1 H), 8.76 (ddd, *J* = 4.7, 1.6, 0.9 Hz, 1 H), 8.39 (dd, *J* = 2.0, 1.3 Hz, 1 H), 8.21 (dd, *J* = 9.2, 2.4 Hz, 1 H), 8.13 (dd, *J* = 9.2, 2.4 Hz, 1 H), 8.02 (ddd, *J* = 7.9, 1.0, 1.0 Hz, 1 H), 7.95 (br s, 1 H), 7.84 (dd, *J* = 7.9, 1.6 Hz, 1 H), 7.70–7.74 (m, 3 H), 7.65 (*J* = 3.9 Hz, 1 H), 4.52 (s, 2 H), 3.09 (s, 3 H). Anal. C, H, N.

#### 4.1.31 *N*-(5-(5-(2-Methyl-1-oxoisoindolin-5-yl)thiophen-2-yl)pyridin-3-yl)benzenesulphonamide (**21**)

Amine **78** was reacted with benzenesulphonyl chloride according to general procedure B to give **21** as a yellow solid (64%); mp 300–303 °C. ^1^H NMR [400 MHz, (CD_3_)_2_SO] δ 10.77 (br s, 1 H), 8.67 (d, *J* = 2.0 Hz, 1 H), 8.21 (d, *J* = 2.4 Hz, 1 H), 7.94 (s, 1 H), 7.75–7.80 (m, 3 H), 7.68–7.76 (m, 3 H), 7.55–7.67 (m, 4 H), 4.52 (s, 2 H), 3.09 (s, 3 H). In this case the product was converted to its sodium salt to give the desired product **21** as a yellow solid (94%). ^1^H NMR [400 MHz, (CD_3_)_2_SO] δ 8.00 (d, *J* = 2.1 Hz, 1 H), 7.90 (s, 1 H), 7.88 (d, *J* = 2.4, 1 H), 7.80 (dd, *J* = 7.9,1.5 Hz, 1 H), 7.71–7.77 (m, *J* = 8.0, 2 H), 7.68 (d, *J* = 8.0, 1 H), 7.63 (d, *J* = 3.8, 1 H), 7.10–7.30 (m, 5 H), 4.51 (s, 2 H), 3.08 (s, 3 H). Anal. C, H, N.

#### 4.1.32 2-Fluoro-*N*-(5-(5-(2-methyl-1-oxoisoindolin-5-yl)thiophen-2-yl)pyridin-3-yl)benzenesulfonamide (**22**)

Amine **78** was reacted with 2-fluorobenzenesulphonyl chloride according to general procedure B to give **22** as a beige solid (82%); mp (CH_2_Cl_2_/MeOH) 289–292 °C. ^1^H NMR [400 MHz, (CD_3_)_2_SO] δ 11.11 (br s, 1 H), 8.67 (d, *J* = 2.0 Hz, 1 H), 8.25 (d, *J* = 2.4 Hz, 1 H), 7.95 (dt, *J* = 7.4, 1.7 Hz, 2 H), 7.83 (dd, *J* = 8.0, 1.5 Hz, 1 H), 7.68–7.76 (m, 4 H), 7.63 (d, *J* = 3.8 Hz, 1 H), 7.38–7.49 (m, 2 H), 4.52 (s, 2 H), 3.09 (s, 3 H). LRMS (APCI^−^) calcd for C_24_H_18_N_3_O_3_FS_2_ 479 (M − H), found 479. Anal. C, H, N.

#### 4.1.33 3-Fluoro-*N*-(5-(5-(2-methyl-1-oxoisoindolin-5-yl)thiophen-2-yl)pyridin-3-yl)benzenesulfonamide (**23**)

Amine **78** was reacted with 3-fluorobenzenesulphonyl chloride according to general procedure B to give **23** as a beige solid (56%); mp (CH_2_Cl_2_/MeOH) 292–294 °C. ^1^H NMR [400 MHz, (CD_3_)_2_SO] δ 10.88 (br s, 1 H), 8.70 (d, *J* = 2.0 Hz, 1 H), 8.22 (d, *J* = 2.3 Hz, 1 H), 7.94 (s, 1 H), 7.83 (dd, *J* = 7.9, 1.5 Hz, 1 H), 7.69–7.75 (m, 3 H), 7.61–7.68 (m, 4 H), 7.50–7.58 (m, 1 H), 4.52 (s, 2 H), 3.09 (s, 3 H). Anal. C, H, N.

#### 4.1.34 4-Fluoro-*N*-(5-(5-(2-methyl-1-oxoisoindolin-5-yl)thiophen-2-yl)pyridin-3-yl)benzenesulfonamide (**24**)

Amine **78** was reacted with 4-fluorobenzenesulphonyl chloride according to general procedure B to give **24** as a yellow solid (86%); mp (CH_2_Cl_2_/MeOH) 272–274 °C. ^1^H NMR [400 MHz, (CD_3_)_2_SO] δ 10.79 (br s, 1 H), 8.69 (d, *J* = 2.0 Hz, 1 H), 8.21 (d, *J* = 2.3 Hz, 1 H), 7.94 (s, 1 H), 7.85–7.92 (m, 2 H), 7.83 (dd, *J* = 7.9, 1.5 Hz, 1 H), 7.68–7.75 (m, 3 H), 7.66 (d, *J* = 3.9 Hz, 1 H), 7.40–7.48 (m, 2 H), 4.52 (s, 2 H), 3.09 (s, 3 H). In this case the product was converted to its sodium salt to give the desired product as a yellow solid (90%). ^1^H NMR [400 MHz, (CD_3_)_2_SO] δ 8.03 (d, *J* = 2.1 Hz, 1 H), 7.90 (s, 1 H), 7.88 (d, *J* = 2.5 Hz, 1 H), 7.73–7.84 (m, 3 H), 7.68 (d, *J* = 7.9 Hz, 1 H), 7.64 (d, *J* = 3.8 Hz, 1 H), 7.37–7.42 (m, 2 H), 7.15–7.23 (m, 2 H), 4.51 (s, 2 H) 3.08 (s, 3 H). Anal. C, H, N.

#### 4.1.35 3,4-Difluoro-*N*-(5-(5-(2-methyl-1-oxoisoindolin-5-yl)thiophen-2-yl)pyridin-3-yl)benzenesulfonamide (**25**)

Amine **78** was reacted with 3,4-difluorobenzenesulphonyl chloride according to general procedure B to give **25** as a yellow solid (18%); mp (CH_2_Cl_2_/MeOH) 282–285 °C. ^1^H NMR [400 MHz, (CD_3_)_2_SO] δ 10.88 (br s, 1 H), 8.72 (d, *J* = 1.4 Hz, 1 H), 8.23 (d, *J* = 2.3 Hz, 1 H), 7.95 (s, 1 H), 7.90 (d, *J* = 8.3 Hz, 1 H), 7.83 (d, *J* = 8.0 Hz, 1 H), 7.66–7.76 (m, 6 H), 4.52 (s, 2 H), 3.09 (s, 3 H). Anal. C, H, N.

#### 4.1.36 2,4,6-Trifluoro-*N*-(5-(5-(2-methyl-1-oxoisoindolin-5-yl)thiophen-2-yl)pyridin-3-yl)benzenesulfonamide (**26**)

Amine **78** was reacted with 2,4,6-trifluorobenzenesulphonyl chloride according to general procedure B to give **26** as a beige solid (16%); mp (CH_2_Cl_2_/MeOH) 272–275 °C. ^1^H NMR [400 MHz, (CD_3_)_2_SO] δ 11.43 (br s, 1 H), 8.28 (d, *J* = 2.3 Hz, 1 H), 7.95 (s, 1 H), 7.83 (dd, *J* = 7.9, 1.5 Hz, 1 H), 7.79 (t, *J* = 2.2 Hz, 1 H), 7.73 (d, *J* = 3.9 Hz, 1 H), 7.71 (d, *J* = 7.9 Hz, 1 H), 7.65 (d, *J* = 3.9 Hz, 1 H), 7.47 (br t, *J* = 9.4 Hz, 2 H), 4.52 (s, 2 H), 3.09 (s, 3 H). Anal. C, H, N.

#### 4.1.37 2-Chloro-*N*-(5-(5-(2-methyl-1-oxoisoindolin-5-yl)thiophen-2-yl)pyridin-3-yl)benzenesulfonamide (**27**)

Amine **78** was reacted with 2-chlorobenzenesulfonyl chloride according to general procedure B to give **27** as a yellow solid (29%); mp (MeOH/CH_2_Cl_2_) 299–303 °C. ^1^H NMR [400 MHz, (CD_3_)_2_SO] δ 11.14 (br s, 1 H), 8.64 (d, *J* = 1.9 Hz, 1 H), 8.26 (d, *J* = 2.3 Hz, 1 H), 8.17 (dd, *J* = 7.3, 1.2 Hz, 1 H), 7.94 (s, 1 H), 7.83 (dd, *J* = 7.9, 1.5 Hz, 1 H), 7.55–7.73 (m, 7 H), 4.52 (s, 2 H), 3.09 (s, 3 H). LRMS (APCI^−^) calcd for C_24_H_17_ClN_3_O_3_S_2_ 495 (M − H), found 495. Anal. C, H, N.

#### 4.1.38 3-Chloro-*N*-(5-(5-(2-methyl-1-oxoisoindolin-5-yl)thiophen-2-yl)pyridin-3-yl)benzenesulfonamide (**28**)

Amine **78** was reacted with 3-chlorobenzenesulfonyl chloride according to general procedure B to give **28** as an orange solid (49%); mp (MeOH/CH_2_Cl_2_) 291–295 °C. ^1^H NMR [400 MHz, (CD_3_)_2_SO] δ 10.88 (br s, 1 H), 8.71 (d, *J* = 2.0 Hz, 1 H), 8.22 (d, *J* = 2.4 Hz, 1 H), 7.95 (d, *J* = 0.7 Hz, 1 H), 7.81–7.86 (m, 2 H), 7.69–7.79 (m, 5 H), 7.67 (d, *J* = 3.9 Hz, 1 H), 7.63 (t, *J* = 7.9 Hz, 1 H), 4.52 (s, 2 H), 3.09 (s, 3 H). LRMS (APCI^−^) calcd for C_24_H_17_ClN_3_O_3_S_2_ 495 (M − H), found 495. Anal. C, H, N.

#### 4.1.39 4-Chloro-*N*-(5-(5-(2-methyl-1-oxoisoindolin-5-yl)thiophen-2-yl)pyridin-3-yl)benzenesulfonamide (**29**)

Amine **78** was reacted with 4-chlorobenzenesulfonyl chloride according to general procedure B to give **29** as a yellow solid (59%); mp (MeOH/CH_2_Cl_2_) 281–284 °C. ^1^H NMR [400 MHz, (CD_3_)_2_SO] δ 10.84 (br s, 1 H), 8.70 (d, *J* = 2.0 Hz, 1 H), 8.21 (d, *J* = 2.3 Hz, 1 H), 7.94 (s, 1 H), 7.79–7.85 (m, 3 H), 7.65–7.74 (m, 6 H), 4.52 (s, 2 H), 3.09 (s, 3 H). LRMS (APCI^−^) calcd for C_24_H_17_ClN_3_O_3_S_2_ 495 (M − H), found 495. Anal. C, H, N. In this case the product was converted to its sodium salt to give the desired product as a yellow solid (77%), mp (EtOH) 240–244 °C. ^1^H NMR [400 MHz, (CD_3_)_2_SO] δ 8.04 (d, *J* = 2.1 Hz, 1 H), 7.91 (d, *J* = 0.8 Hz, 1 H), 7.89 (d, *J* = 2.4 Hz, 1 H), 7.80 (dd, *J* = 7.9, 1.5 Hz, 1 H), 7.73 (d, *J* = 8.6 Hz, 2 H), 7.68 (d, *J* = 7.8 Hz, 1 H), 7.64 (d, *J* = 3.8 Hz, 1 H), 7.43 (d, *J* = 8.6 Hz, 2 H), 7.41 (d, *J* = 3.8 Hz, 1 H), 7.39 (t, *J* = 2.3 Hz, 1 H), 4.51 (s, 2 H), 3.08 (s, 3 H). Anal. C, H, N.

#### 4.1.40 3,4-Dichloro-*N*-(5-(5-(2-methyl-1-oxoisoindolin-5-yl)thiophen-2-yl)pyridin-3-yl)benzenesulfonamide (**30**)

Amine **78** was reacted with 3,4-dichlorobenzenesulfonyl chloride according to general procedure B to give **30** as an orange-brown solid (15%); mp (MeOH/CH_2_Cl_2_) 256–259 °C. ^1^H NMR [400 MHz, (CD_3_)_2_SO] δ 10.92 (br s, 1 H), 8.72 (d, *J* = 2.0 Hz, 1 H), 8.23 (d, *J* = 2.4 Hz, 1 H), 8.03 (d, *J* = 2.2 Hz, 1 H), 7.94 (s, 1 H), 7.89 (d, *J* = 8.5 Hz, 1 H), 7.83 (dd, *J* = 8.0, 1.6 Hz, 1 H), 7.70–7.77 (m, 4 H), 7.69 (d, *J* = 3.9 Hz, 1 H), 4.52 (s, 2 H), 3.09 (s, 3 H). HRMS (ESI^−^) calcd for C_24_H_16_Cl_2_N_3_O_3_S_2_ 528.0016 (M − H), found 528.0048.

#### 4.1.41 2,4-Dichloro-*N*-(5-(5-(2-methyl-1-oxoisoindolin-5-yl)thiophen-2-yl)pyridin-3-yl)benzenesulfonamide (**31**)

Amine **78** was reacted with 2,4-dichlorobenzenesulphonyl chloride according to general procedure B to give **31** as a yellow solid (55%); mp (CH_2_Cl_2_/MeOH) 282–285 °C. ^1^H NMR [400 MHz, (CD_3_)_2_SO] δ 11.20 (br s, 1 H), 8.67 (d, *J* = 2.0 Hz, 1 H), 8.26 (d, *J* = 2.4 Hz, 1 H), 8.14 (d, *J* = 8.6 Hz, 1 H), 7.96 (s, 1 H), 7.91 (d, *J* = 2.0 Hz, 1 H), 7.83 (dd, *J* = 8.0, 1.5 Hz, 1 H), 7.65–7.74 (m, 4 H), 7.64 (d, *J* = 3.9 Hz, 1 H), 4.52 (s, 2 H), 3.09 (s, 3 H). In this case the product was converted to its sodium salt to give the desired product as a beige solid (89%). ^1^H NMR [400 MHz, (CD_3_)_2_SO] δ 8.08 (d, *J* = 2.1 Hz, 1 H), 8.01 (d, *J* = 8.4 Hz, 1 H), 7.88–7.92 (m, 2 H), 7.80 (dd, *J* = 7.9, 1.5 Hz, 1 H), 7.68 (d, *J* = 8.0, 1 H), 7.64 (d, *J* = 3.8, 1 H), 7.54 (d, *J* = 2.1, 1 H), 7.46 (dd, *J* = 8.4, 2.2 Hz, 1 H), 7.41 (d, *J* = 3.8 Hz, 1 H), 7.37 (t, *J* = 2.3 Hz, 1 H), 4.51 (s, 2 H), 3.08 (s, 3 H). Anal. C, H, N.

#### 4.1.42 2-Bromo-*N*-(5-(5-(2-methyl-1-oxoisoindolin-5-yl)thiophen-2-yl)pyridin-3-yl)benzenesulfonamide (**32**)

Amine **78** was reacted with 2-bromobenzenesulfonyl chloride according to general procedure B to give **32** as an orange solid (30%); mp (MeOH/CH_2_Cl_2_) 289–293 °C. ^1^H NMR [400 MHz, (CD_3_)_2_SO] δ 11.15 (br s, 1 H), 8.63 (s, 1 H), 8.26 (d, *J* = 2.3 Hz, 1 H), 8.20 (dd, *J* = 7.9, 1.7 Hz, 1 H), 7.94 (s, 1 H), 7.80–7.88 (m, 2 H), 7.68–7.74 (m, 2 H), 7.67 (t, *J* = 2.2 Hz, 1 H), 7.59–7.65 (m, 2 H), 7.54 (dt, *J* = 7.6, 1.7 Hz, 1 H), 4.52 (s, 2 H), 3.09 (s, 3 H). LRMS (APCI^−^) calcd for C_24_H_17_BrN_3_O_3_S_2_ 539 (M − H), found 539. Anal. C, H, N.

#### 4.1.43 3-Bromo-*N*-(5-(5-(2-methyl-1-oxoisoindolin-5-yl)thiophen-2-yl)pyridin-3-yl)benzenesulfonamide (**33**)

Amine **78** was reacted with 3-bromobenzenesulfonyl chloride according to general procedure B to give **33** as a **y**ellow-orange solid (30%); mp (MeOH/CH_2_Cl_2_) 303–307 °C. ^1^H NMR [400 MHz, (CD_3_)_2_SO] δ 10.87 (br s, 1 H), 8.71 (d, *J* = 1.9 Hz, 1 H), 8.21 (d, *J* = 2.3 Hz, 1 H), 7.98 (t, *J* = 1.8 Hz, 1 H), 7.95 (d, *J* = 0.7 Hz, 1 H), 7.78–7.90 (m, 3 H), 7.69–7.75 (m, 3 H), 7.67 (d, *J* = 3.9 Hz, 1 H), 7.56 (t, *J* = 8.0 Hz, 1 H), 4.52 (s, 2 H), 3.09 (s, 3 H). HRMS (ESI^+^) calcd for C_24_H_18_BrN_3_O_3_S_2_Na 561.9865 (M + Na^+^), found 561.9862. Anal. C, H, N.

#### 4.1.44 4-Bromo-*N*-(5-(5-(2-methyl-1-oxoisoindolin-5-yl)thiophen-2-yl)pyridin-3-yl)benzenesulfonamide (**34**)

Amine **78** was reacted with 4-bromobenzenesulfonyl chloride according to general procedure B to give **34** as a yellow solid (60%); mp (MeOH/CH_2_Cl_2_) 276–279 °C. ^1^H NMR [400 MHz, (CD_3_)_2_SO] δ 10.84 (br s, 1 H), 8.69 (d, *J* = 2.0 Hz, 1 H), 8.21 (d, *J* = 2.4 Hz, 1 H), 7.94 (d, *J* = 0.8 Hz, 1 H), 7.80–7.86 (m, 3 H), 7.69–7.76 (m, 5 H), 7.66 (d, *J* = 3.9 Hz, 1 H), 4.52 (s, 2 H), 3.09 (s, 3 H). LRMS (APCI^−^) calcd for C_24_H_17_BrN_3_O_3_S_2_ 539 (M − H), found 539. Anal. C, H, N.

#### 4.1.45 2,4-Dibromo-*N*-(5-(5-(2-methyl-1-oxoisoindolin-5-yl)thiophen-2-yl)pyridin-3-yl)benzenesulfonamides (**35**)

Amine **78** was reacted with 2,4-dibromobenzenesulfonyl chloride according to general procedure B to give **35** as a cream solid (81%); mp (MeOH/CH_2_Cl_2_) 276–279 °C. ^1^H NMR [400 MHz, (CD_3_)_2_SO] δ 11.23 (br s, 1 H), 8.66 (d, *J* = 1.9 Hz, 1 H), 8.25 (d, *J* = 2.4 Hz, 1 H), 8.16 (d, *J* = 1.9 Hz, 1 H), 8.08 (d, *J* = 8.5 Hz, 1 H), 7.94 (d, *J* = 0.7 Hz, 1 H), 7.81–7.87 (m, 2 H), 7.67–7.74 (m, 2 H), 7.65 (t, *J* = 2.2 Hz, 1 H), 7.63 (d, *J* = 3.8 Hz, 1 H), 4.52 (s, 2 H), 3.09 (s, 3 H). LRMS (APCI^−^) calcd for C_24_H_17_Br_2_N_3_O_3_S_2_ 617, 619, 621 (M), found 617, 619, 621. Anal. C, H, N.

#### 4.1.46 4-Iodo-*N*-(5-(5-(2-methyl-1-oxoisoindolin-5-yl)thiophen-2-yl)pyridin-3-yl)benzenesulfonamides (**36**)

Amine **78** was reacted with 4-iodobenzenesulfonyl chloride according to general procedure B to give **36** as a pale yellow solid (59%); mp (CH_2_Cl_2_/Et_2_O) 295–298 °C. ^1^H NMR [400 MHz, (CD_3_)_2_SO] δ 10.80 (br s, 1 H), 8.70 (d, *J* = 2.0 Hz, 1 H), 8.20 (d, *J* = 2.4 Hz, 1 H), 7.99 (d, *J* = 8.6 Hz, 2 H), 7.95 (d, *J* = 0.70 Hz, 1 H), 7.84 (dd, *J* = 8.0, 1.5 Hz, 1 H), 7.70–7.74 (m, 2 H), 7.68 (t, *J* = 2.2 Hz, 1 H), 7.66 (d, *J* = 3.9 Hz, 1 H), 7.56 (d, *J* = 8.6 Hz, 2 H), 4.52 (s, 2 H), 3.09 (s, 3 H). LRMS (APCI^−^) calcd for C_24_H_18_IN_3_O_3_S_2_ 587 (M), found 587. Anal. C, H, N.

#### 4.1.47 2-Methoxy-*N*-(5-(5-(2-methyl-1-oxoisoindolin-5-yl)thiophen-2-yl)pyridin-3-yl)benzenesulfonamide (**37**)

Amine **78** reacted with 2-methoxybenzenesulfonyl chloride according to general procedure B to give **37** as a yellow solid (26%); mp (MeOH/CH_2_Cl_2_) 273–276 °C. ^1^H NMR [400 MHz, (CD_3_)_2_SO] δ 10.49 (br s, 1 H), 8.61 (d, *J* = 2.0 Hz, 1 H), 8.24 (d, *J* = 2.4 Hz, 1 H), 7.94 (s, 1 H), 7.86 (dd, *J* = 7.9, 1.7 Hz, 1 H), 7.82 (dd, *J* = 8.0, 1.6 Hz, 1 H), 7.67–7.73 (m, 3 H), 7.56–7.62 (m, 2 H), 7.19 (d, *J* = 7.9 Hz, 1 H), 7.08 (dd, *J* = 7.6, 0.7 Hz, 1 H), 4.52 (s, 2 H), 3.88 (s, 3 H), 3.09 (s, 3 H). LRMS (APCI^−^) calcd for C_25_H_20_N_3_O_4_S_2_ 491 (M − H), found 491. Anal. C, H, N.

#### 4.1.48 3-Methoxy-*N*-(5-(5-(2-methyl-1-oxoisoindolin-5-yl)thiophen-2-yl)pyridin-3-yl)benzenesulfonamide (**38**)

Amine **78** was reacted with 3-methoxybenzenesulfonyl chloride according to general procedure B to give **38** as a pale yellow solid (33%); mp (MeOH/CH_2_Cl_2_) 278–280 °C. ^1^H NMR [400 MHz, (CD_3_)_2_SO] δ 10.76 (br s, 1 H), 8.67 (d, *J* = 2.0 Hz, 1 H), 8.21 (d, *J* = 2.3 Hz, 1 H), 7.94 (d, *J* = 0.7 Hz, 1 H), 7.83 (dd, *J* = 8.0, 1.5 Hz, 1 H), 7.68–7.75 (m, 3 H), 7.65 (d, *J* = 3.9 Hz, 1 H), 7.51 (t, *J* = 8.0 Hz, 1 H), 7.36–7.41 (m, 1 H), 7.32 (t, *J* = 2.1 Hz, 1 H), 7.21 (ddd, *J* = 8.3, 2.6, 0.8 Hz, 1 H), 4.52 (s, 2 H), 3.79 (s, 3 H), 3.09 (s, 3 H). HRMS (ESI^+^) calcd for C_25_H_22_N_3_O_4_S_2_ 492.1046 (MH^+^), found 492.1033. Anal. C, H, N.

#### 4.1.49 4-Methoxy-*N*-(5-(5-(2-methyl-1-oxoisoindolin-5-yl)thiophen-2-yl)pyridin-3-yl)benzenesulfonamide (**39**)

Amine **78** was reacted with 4-methoxybenzenesulfonyl chloride according to general procedure B to give **39** as a dark yellow solid (37%); mp (MeOH/CH_2_Cl_2_) 254–258 °C. ^1^H NMR [400 MHz, (CD_3_)_2_SO] δ 10.62 (br s, 1 H), 8.66 (d, *J* = 1.8 Hz, 1 H), 8.20 (d, *J* = 2.2 Hz, 1 H), 7.94 (s, 1 H), 7.83 (d, *J* = 8.3 Hz, 1 H), 7.76 (d, *J* = 8.9 Hz, 2 H), 7.67–7.74 (m, 3 H), 7.64 (d, *J* = 3.8 Hz, 1 H), 7.10 (d, *J* = 8.9 Hz, 2 H), 4.52 (s, 2 H), 3.79 (s, 3 H), 3.09 (s, 3 H). HRMS (ESI^+^) calcd for C_25_H_22_N_3_O_4_S_2_ 492.1046 (MH^+^), found 492.1033.

#### 4.1.50 3,4-Dimethoxy-*N*-(5-(5-(2-methyl-1-oxoisoindolin-5-yl)thiophen-2-yl)pyridin-3-yl)benzenesulfonamide, sodium salt (**40**)

Amine **78** was reacted with 3,4-dimethoxybenzenesulfonyl chloride according to general procedure B to give **40** as a pale orange solid (31%). ^1^H NMR [400 MHz, (CD_3_)_2_SO] δ 10.57 (br s, 1 H), 8.66 (d, *J* = 2.0 Hz, 1 H), 8.21 (d, *J* = 2.4 Hz, 1 H), 7.94 (d, *J* = 0.7 Hz, 1 H), 7.83 (dd, *J* = 7.9, 1.5 Hz, 1 H), 7.69–7.75 (m, 3 H), 7.65 (d, *J* = 3.9 Hz, 1 H), 7.39 (dd, *J* = 8.5, 2.2 Hz, 1 H), 7.32 (d, *J* = 2.2 Hz, 1 H), 7.10 (d, *J* = 8.6 Hz, 1 H), 4.52 (s, 2 H), 3.79 (s, 3 H), 3.78 (s, 3 H), 3.09 (s, 3 H). LRMS (APCI^−^) calcd for C_26_H_22_N_3_O_5_S_2_ 521 (M − H), found 521. In this case, the product was also converted to the sodium salt according to general procedure E to give the title compound as a yellow solid (96%); mp (EtOH) 240–244 °C. ^1^H NMR [400 MHz, (CD_3_)_2_SO] δ 8.00 (d, *J* = 2.1 Hz, 1 H), 7.89 (d, *J* = 0.8 Hz, 1 H), 7.86 (d, *J* = 2.5 Hz, 1 H), 7.79 (dd, *J* = 8.0, 1.6 Hz, 1 H), 7.68 (d, *J* = 7.9 Hz, 1 H), 7.64 (d, *J* = 3.8 Hz, 1 H), 7.37–7.41 (m, 2 H), 7.28–7.32 (m, 2 H), 6.92 (d, *J* = 8.1 Hz, 1 H), 4.51 (s, 2 H), 3.76 (s, 3 H), 3.73 (s, 3 H), 3.08 (s, 3 H). Anal. C, H, N.

#### 4.1.51 *N*-(5-(5-(2-Methyl-1-oxoisoindolin-5-yl)thiophen-2-yl)pyridin-3-yl)-2-(trifluoromethoxy)benzenesulfonamide (**41**)

Amine **78** was reacted with 2-trifluoromethoxybenzenesulphonyl chloride according to general procedure B to give **41** as a pale yellow solid (42%); mp (CH_2_Cl_2_/MeOH) 251–254 °C. ^1^H NMR [400 MHz, (CD_3_)_2_SO] δ 11.03 (br s, 1 H), 8.67 (d, *J* = 1.7 Hz, 1 H), 8.23 (d, *J* = 2.3 Hz, 1 H), 8.09 (dd, *J* = 7.8, 1.6 Hz, 1 H), 7.94 (s, 1 H), 7.75–7.85 (m, 2 H), 7.68–7.74 (m, 3 H), 7.63 (d, *J* = 3.8 Hz, 1 H), 7.55–7.62 (m, 2 H), 4.52 (s, 2 H), 3.09 (s, 3 H). Anal. C, H, N.

#### 4.1.52 *N*-(5-(5-(2-Methyl-1-oxoisoindolin-5-yl)thiophen-2-yl)pyridin-3-yl)-3-(trifluoromethoxy)benzenesulfonamide (**42**)

Amine **78** was reacted with 3-trifluoromethoxybenzenesulphonyl chloride according to general procedure B to give **42** as a light brown solid (22%); mp (MeOH/CH_2_Cl_2_) 230–232 °C. ^1^H NMR [400 MHz, (CD_3_)_2_SO] δ 10.90 (br s, 1 H), 8.70 (d, *J* = 2.0 Hz, 1 H), 8.20 (d, *J* = 2.3 Hz, 1 H), 7.94 (s, 1 H), 7.80–7.87 (m, 2 H), 7.67–7.79 (m, 6 H), 7.65 (d, *J* = 3.8 Hz, 1 H), 4.52 (s, 2 H), 3.09 (s, 3 H). HRMS (APCI^+^) calcd for C_25_H_18_F_3_N_3_O_4_S_2_ 546.0764 (MH^+^), found 546.0747.

#### 4.1.53 *N*-(5-(5-(2-Methyl-1-oxoisoindolin-5-yl)thiophen-2-yl)pyridin-3-yl)-4-(trifluoromethoxy)benzenesulfonamide (**43**)

Amine **78** was reacted with 4-trifluoromethoxybenzenesulphonyl chloride according to general procedure B to give **43** as a pale yellow solid (41%); mp (CH_2_Cl_2_/MeOH) 293–296 °C. ^1^H NMR [400 MHz, (CD_3_)_2_SO] δ 10.88 (br s, 1 H), 8.70 (d, *J* = 2.0 Hz, 1 H), 8.22 (d, *J* = 2.3 Hz, 1 H), 7.92–7.98 (m, 3 H), 7.82 (dd, *J* = 8.0, 1.5 Hz, 1 H), 7.72 (m, 3 H), 7.66 (d, *J* = 3.9 Hz, 1 H), 7.57–7.63 (m, 2 H), 4.52 (s, 2 H), 3.09 (s, 3 H). Anal. C, H, N.

#### 4.1.54 *N*-(5-(5-(2-Methyl-1-oxoisoindolin-5-yl)thiophen-2-yl)pyridin-3-yl)-2-(trifluoromethyl)benzenesulfonamide (**44**)

Amine **78** was reacted with 2-(trifluoromethyl)benzenesulfonyl chloride according to general procedure B to give **44** as a pale yellow solid (67%); mp (CH_2_Cl_2_/Et_2_O) 271–275 °C. ^1^H NMR [400 MHz, (CD_3_)_2_SO] δ 11.15 (br s, 1 H), 8.68 (d, *J* = 1.8 Hz, 1 H), 8.25 (d, *J* = 2.3 Hz, 1 H), 8.21 (d, *J* = 7.7 Hz, 1 H), 8.03 (d, *J* = 7.4 Hz, 1 H), 7.84–7.96 (m, 3 H), 7.82 (dd, *J* = 8.0, 1.4 Hz, 1 H), 7.68–7.74 (m, 3 H), 7.63 (d, *J* = 3.8 Hz, 1 H), 4.52 (s, 2 H), 3.09 (s, 3 H). LRMS (APCI^−^) calcd for C_25_H_18_F_3_N_3_O_3_S_2_ 529 (M), found 529. Anal. C, H, N.

#### 4.1.55 *N*-(5-(5-(2-Methyl-1-oxoisoindolin-5-yl)thiophen-2-yl)pyridin-3-yl)-3-(trifluoromethyl)benzenesulfonamides (**45**)

Amine **78** was reacted with 3-(trifluoromethyl)benzenesulfonyl chloride according to general procedure B to give **45** as a pale yellow solid (65%); mp (1,4-dioxane) 262–265 °C. ^1^H NMR [400 MHz, (CD_3_)_2_SO] δ 10.92 (br s, 1 H), 8.71 (d, *J* = 2.0 Hz, 1 H), 8.20 (d, *J* = 2.3 Hz, 1 H), 8.05–8.12 (m, 3 H), 7.93 (d, *J* = 0.7 Hz, 1 H), 7.86 (d, *J* = 7.9 Hz, 1 H), 7.82 (dd, *J* = 7.9, 1.6 Hz, 1 H), 7.68–7.74 (m, 3 H), 7.66 (d, *J* = 3.8 Hz, 1 H), 4.52 (s, 2 H), 3.09 (s, 3 H). LRMS (APCI^−^) calcd for C_25_H_18_F_3_N_3_O_3_S_2_ 529 (M), found 529. Anal. C, H, N.

#### 4.1.56 *N*-(5-(5-(2-Methyl-1-oxoisoindolin-5-yl)thiophen-2-yl)pyridin-3-yl)-4-(trifluoromethyl)benzenesulfonamide (**46**)

Amine **78** was reacted with 4-trifluoromethylbenzenesulphonyl chloride according to general procedure B to give **46** as a pink solid (55%); mp (CH_2_Cl_2_/MeOH) 282–284 °C. ^1^H NMR [400 MHz, (CD_3_)_2_SO] δ 10.99 (br s, 1 H), 8.72 (d, *J* = 2.0 Hz, 1 H), 8.22 (d, *J* = 2.3 Hz, 1 H), 7.97–8.07 (m, 4 H), 7.94 (s, 1 H), 7.83 (dd, *J* = 8.0, 1.6 Hz, 1 H), 7.68–7.76 (m, 3 H), 7.67 (d, *J* = 3.9 Hz, 1 H), 4.52 (s, 2 H), 3.09 (s, 3 H). Anal. C, H, N.

#### 4.1.57 *N*-(5-(5-(2-Methyl-1-oxoisoindolin-5-yl)thiophen-2-yl)pyridin-3-yl)-3,5-bis(trifluoromethyl)benzenesulphonamide (**47**)

Amine **78** was reacted with 3,5-bis(trifluoromethyl)benzene-1-sulphonyl chloride according to general procedure B to give **47** as a beige solid (63%); mp (MeOH/CH_2_Cl_2_) 299–302 °C. ^1^H NMR [400 MHz, (CD_3_)_2_SO] δ 11.04 (br s, 1 H), 8.75 (d, *J* = 2.0 Hz, 1 H), 8.53 (s, 1 H), 8.37 (s, 2 H), 8.22 (d, *J* = 2.4 Hz, 1 H), 7.93 (s, 1 H), 7.82 (dd, *J* = 7.9, 1.4 Hz, 1 H), 7.72–7.76 (m, 2 H), 7.71 (d, *J* = 7.9 Hz, 1 H), 7.68 (d, *J* = 3.9 Hz, 1 H), 4.52 (s, 2 H), 3.09 (s, 3 H). Anal. C, H, N.

#### 4.1.58 2-Cyano-*N*-(5-(5-(2-methyl-1-oxoisoindolin-5-yl)thiophen-2-yl)pyridin-3-yl)benzenesulfonamide (**48**)

Amine **78** was reacted with 2-cyanobenzenesulfonyl chloride according to general procedure B to give **48** as a pale mustard-yellow solid (43%); mp (MeOH/CH_2_Cl_2_) >310 °C. ^1^H NMR [400 MHz, (CD_3_)_2_SO] δ 11.16 (br s, 1 H), 8.95 (d, *J* = 1.8 Hz, 1 H), 8.89 (d, *J* = 1.5 Hz, 1 H), 8.57 (t, *J* = 2.1 Hz, 1 H), 8.47 (d, *J* = 7.4 Hz, 1 H), 8.13 (d, *J* = 7.0 Hz, 1 H), 7.90–8.00 (m, 3 H), 7.85 (dd, *J* = 7.9, 1.3 Hz, 1 H), 7.79 (d, *J* = 3.9 Hz, 1 H), 7.76 (d, *J* = 3.9 Hz, 1 H), 7.72 (d, *J* = 8.0 Hz, 1 H), 4.53 (s, 2 H), 3.09 (s, 3 H). LRMS (APCI^−^) calcd for C_25_H_18_N_4_O_3_S_2_ 486 (M), found 486. Anal. C, H, N. In this case the product was also converted to its sodium salt according to general procedure E to give the desired product as a pale yellow solid (100%). ^1^H NMR [400 MHz, (CD_3_)_2_SO] δ 8.47 (t, *J* = 2.5 Hz, 2 H), 8.32 (t, *J* = 2.2 Hz, 1 H), 7.91–7.96 (m, 2 H), 7.85 (dd, *J* = 8.0, 1.4 Hz, 1 H), 7.67–7.73 (m, 3 H), 7.68 (d, *J* = 3.9 Hz, 1 H), 7.55–7.61 (m, 2 H), 4.52 (s, 2 H), 3.09 (s, 3 H).

#### 4.1.59 3-Cyano-*N*-(5-(5-(2-methyl-1-oxoisoindolin-5-yl)thiophen-2-yl)pyridin-3-yl)benzenesulfonamide (**49**)

Amine **78** was reacted with 3-cyanobenzenesulfonyl chloride according to general procedure B to give **49** as a cream solid (39%); mp (CH_2_Cl_2_/Et_2_O) 282–285 °C. ^1^H NMR [400 MHz, (CD_3_)_2_SO] δ 10.96 (br s, 1 H), 8.71 (d, *J* = 2.0 Hz, 1 H), 8.30 (t, *J* = 1.5 Hz, 1 H), 8.22 (d, *J* = 2.3 Hz, 1 H), 8.15 (dt, *J* = 7.9, 1.2 Hz, 1 H), 8.10 (ddd, *J* = 8.1, 1.9, 1.1 Hz, 1 H), 7.95 (d, *J* = 0.8 Hz, 1 H), 7.79–7.86 (m, 2 H), 7.67–7.74 (m, 4 H), 4.51 (s, 2 H), 3.09 (s, 3 H). LRMS (APCI^+^) calcd for C_25_H_19_N_4_O_3_S_2_ 487 (MH^+^), found 487. Anal. C, H, N.

#### 4.1.60 4-Cyano-*N*-(5-(5-(2-methyl-1-oxoisoindolin-5-yl)thiophen-2-yl)pyridin-3-yl)benzenesulfonamide (**50**)

Amine **78** was reacted with 4-cyanobenzenesulphonyl chloride according to general procedure B to give **50** as a yellow solid (10%); mp (CH_2_Cl_2_/MeOH) 282–285 °C. ^1^H NMR [400 MHz, (CD_3_)_2_SO] δ 11.02 (br s, 1 H), 8.71 (d, *J* = 2.0 Hz, 1 H), 8.21 (d, *J* = 2.4 Hz, 1 H), 8.08 (d, *J* = 8.6 Hz, 2 H), 7.98 (d, *J* = 8.6 Hz, 2 H), 7.95 (s, 1 H), 7.83 (dd, *J* = 8.0, 1.6 Hz, 1 H), 7.74 (d, *J* = 4.0 Hz, 1 H), 7.72 (s, 1 H), 7.71 (d, *J* = 8.4 Hz, 1 H), 7.68 (d, *J* = 3.9 Hz, 1 H), 4.52 (s, 2 H), 3.09 (s, 3 H). Anal. C, H, N.

#### 4.1.61 Methyl 2-(*N*-(5-(5-(2-methyl-1-oxoisoindolin-5-yl)thiophen-2-yl)pyridin-3-yl)sulfamoyl)benzoate (**51**)

Amine **78** was reacted with methyl 2-(chlorosulfonyl)benzoate according to general procedure B to give **51** as a pale orange solid (23%); mp (CH_2_Cl_2_/Et_2_O) 209–212 °C. ^1^H NMR [400 MHz, (CD_3_)_2_SO] δ 10.74 (br s, 1 H), 8.66 (d, *J* = 2.0 Hz, 1 H), 8.22 (d, *J* = 2.4 Hz, 1 H), 7.95–7.99 (m, 1 H), 7.93 (br d, *J* = 0.7 Hz, 1 H), 7.82 (dd, *J* = 8.0, 1.6 Hz, 1 H), 7.68–7.76 (m, 5 H), 7.63–7.67 (m, 2 H), 4.52 (s, 2 H), 3.85 (s, 3 H), 3.09 (s, 3 H). LRMS (APCI^+^) calcd for C_26_H_22_N_3_O_5_S_2_ 520 (MH^+^), found 520. Anal. C, H, N.

#### 4.1.62 Methyl 3-(*N*-(5-(5-(2-methyl-1-oxoisoindolin-5-yl)thiophen-2-yl)pyridin-3-yl)sulfamoyl)benzoate (**52**)

Amine **78** was reacted with methyl 3-(chlorosulfonyl)benzoate according to general procedure B giving **52** as a beige solid (24%); mp (CH_2_Cl_2_/MeOH) 250–254 °C. ^1^H NMR [400 MHz, (CD_3_)_2_SO] δ 10.92 (s, 1 H), 8.70 (d, *J* = 8.7 Hz, 1 H), 8.38 (dd, *J* = 1.6, 1.6 Hz, 1 H), 8.19–8.21 (m, 2 H), 8.07 (ddd, *J* = 7.9, 1.9, 1.1 Hz, 1 H), 7.94 (br s, 1 H), 7.77 (d, *J* = 7.9 Hz, 1 H), 7.70–7.75 (m, 3 H), 7.66 (d, *J* = 3.4 Hz, 1 H), 4.52 (s, 2 H), 3.87 (s, 3 H), 3.09 (s, 3 H). HRMS (ESI^+^) calcd for C_26_H_22_N_3_O_5_S_2_ 520.0995 (MH^+^), found 520.1004.

#### 4.1.63 Methyl 4-(*N*-(5-(5-(2-methyl-1-oxoisoindolin-5-yl)thiophen-2-yl)pyridin-3-yl)sulfamoyl)benzoate (**53**)

Amine **78** was reacted with methyl 4-(chlorosulfonyl)benzoate according to general procedure B to give **53** as a pale yellow solid (60%); mp (CH_2_Cl_2_/Et_2_O) 269–272 °C. ^1^H NMR [400 MHz, (CD_3_)_2_SO] δ 10.95 (br s, 1 H), 8.67 (d, *J* = 1.9 Hz, 1 H), 8.19 (d,*J* = 2.3 Hz, 1 H), 8.13 (d, *J* = 8.6 Hz, 2 H), 7.92–7.97 (m, 3 H), 7.82 (dd, *J* = 8.0, 1.6 Hz, 1 H), 7.68–7.73 (m, 3 H), 7.65 (d, *J* = 3.9 Hz, 1 H), 4.52 (s, 2 H), 3.85 (s, 3 H), 3.09 (s, 3 H). LRMS (APCI^+^) calcd for C_26_H_22_N_3_O_5_S_2_ 520 (MH^+^), found 520. Anal. C, H, N.

#### 4.1.64 Ethyl 4-(*N*-(5-(5-(2-methyl-1-oxoisoindolin-5-yl)thiophen-2-yl)pyridin-3-yl)sulfamoyl)benzoate (**54**)

Amine **78** was reacted with ethyl 4-(chlorosulfonyl)benzoate according to general procedure B to give **54** as a pale yellow solid (52%); mp (CH_2_Cl_2_/Et_2_O) 272–275 °C. ^1^H NMR [400 MHz, (CD_3_)_2_SO] δ 10.93 (br s, 1 H), 8.69 (d, *J* = 2.0 Hz, 1 H), 8.20 (d, *J* = 2.4 Hz, 1 H), 8.13 (d, *J* = 8.6 Hz, 2 H), 7.93–7.98 (m, 3 H), 7.83 (dd, *J* = 8.0, 1.6 Hz, 1 H), 7.69–7.74 (m, 3 H), 7.66 (d, *J* = 3.9 Hz, 1 H), 4.52 (s, 2 H), 4.31 (q, *J* = 7.1 Hz, 2 H), 3.09 (s, 3 H), 1.29 (t, *J* = 7.1 Hz, 3 H). LRMS (APCI^+^) calcd for C_27_H_24_N_3_O_5_S_2_ 534 (MH^+^), found 534. Anal. C, H, N.

#### 4.1.65 4-(*N*-(5-(5-(2-Methyl-1-oxoisoindolin-5-yl)thiophen-2-yl)pyridin-3-yl)sulfamoyl)benzoic acid (**55**)

Amine **78** was reacted with 4-chlorosulphonyl benzoic acid according to general procedure B to give **55** as a pale pink solid (6%); mp (MeOH/CH_2_Cl_2_) >310 °C. ^1^H NMR [400 MHz, (CD_3_)_2_SO] δ 13.46 (v br s, 1 H), 10.91 (v br s, 1 H), 8.68 (d, *J* = 1.9 Hz, 1 H), 8.21 (d, *J* = 2.3 Hz, 1 H), 8.11 (d, *J* = 8.6 Hz, 2 H), 7.91–7.95 (m, 3 H), 7.83 (dd, *J* = 8.0, 1.5 Hz, 1 H), 7.69–7.73 (m, 3 H), 7.65 (d, *J* = 3.9 Hz, 1 H), 4.52 (s, 2 H), 3.09 (s, 3 H). LRMS (APCI^−^) calcd for C_25_H_19_N_3_O_5_S_2_ 505 (M), found 505. Anal. C, H, N.

#### 4.1.66 *N*-(5-(5-(2-Methyl-1-oxoisoindolin-5-yl)thiophen-2-yl)pyridin-3-yl)-2-(methylsulfonyl)benzenesulfonamide (**56**)

Amine **78** was reacted with 2-(methanesulfonyl)benzenesulfonyl chloride according to general procedure B to give **56** as a pale orange solid (77%); mp (Et_2_O/CH_2_Cl_2_) 269–272 °C. ^1^H NMR [400 MHz, (CD_3_)_2_SO] δ 10.22 (br s, 1 H), 8.68 (d, *J* = 1.5 Hz, 1 H), 8.22–8.27 (m, 2 H), 8.14–8.18 (m, 1 H), 7.89–7.97 (m, 3 H), 7.82 (dd, *J* = 8.0, 1.5 Hz, 1 H), 7.76 (t, *J* = 2.2 Hz, 1 H), 7.68–7.73 (m, 2 H), 7.65 (d, *J* = 3.9 Hz, 1 H), 4.52 (s, 2 H), 3.53 (s, 3 H), 3.09 (s, 3 H). LRMS (APCI^−^) calcd for C_25_H_21_N_3_O_5_S_3_ 539 (M), found 539. Anal. C, H, N.

#### 4.1.67 Sodium (5-(5-(2-methyl-1-oxoisoindolin-5-yl)thiophen-2-yl)pyridin-3-yl)((4-(methylsulfonyl)phenyl)sulfonyl)amide (**57**)

Amine **78** was reacted with 4-(methanesulfonyl)benzenesulfonyl chloride according to general procedure B and the resulting crude product converted directly to the sodium salt according to general procedure E. The salt was recrystallised from EtOH to give **57** as a cream solid (27%); mp (EtOH) > 300 °C. ^1^H NMR [400 MHz, (CD_3_)_2_SO] δ 8.07 (d, *J* = 2.1 Hz, 1 H), 7.91–7.98 (m, 5 H), 7.90 (d, *J* = 0.7 Hz, 1 H), 7.81 (dd, *J* = 8.0, 1.5 Hz, 1 H), 7.68 (d, *J* = 8.2 Hz, 1 H), 7.65 (d, *J* = 3.8 Hz, 1 H), 7.42–7.45 (m, 2 H), 4.52 (s, 2 H), 3.19 (s, 3 H), 3.09 (s, 3 H). LRMS (APCI^−^) calcd for C_25_H_21_N_3_O_5_S_3_ 539 (M − Na), found 539. HRMS (ESI^+^) calcd for C_25_H_21_N_3_NaO_5_S_3_ 562.0536 (MH^+^), found 562.0522.

#### 4.1.68 *N*-(5-(5-(2-Methyl-1-oxoisoindolin-5-yl)thiophen-2-yl)pyridin-3-yl)-2-nitrobenzenesulfonamide (**58**)

Amine **78** was reacted with 2-nitrobenzenesulfonyl chloride according to general procedure B to give **58** as a yellow solid (32%); mp (MeOH/CH_2_Cl_2_) 270–273 °C. ^1^H NMR [400 MHz, (CD_3_)_2_SO] δ 11.21 (bs, 1 H), 8.71 (d, *J* = 1.9 Hz, 1 H), 8.26 (d, *J* = 2.4 Hz, 1 H), 8.06–8.10 (m, 1 H), 7.98–8.01 (m, 1 H), 7.94 (d, *J* = 0.8 Hz, 1 H), 7.86–7.90 (m, 2 H), 7.83 (dd, *J* = 8.0, 1.6 Hz, 1 H), 7.72–7.75 (m, 2 H), 7.71 (d, *J* = 8.0 Hz, 1 H), 7.66 (d, *J* = 3.9 Hz, 1 H), 4.52 (s, 2 H), 3.09 (s, 3 H). LRMS (APCI^−^) calcd for C_24_H_17_N_4_O_5_S_2_ 506 (M − H), found 506. Anal. C, H, N.

#### 4.1.69 *N*-(5-(5-(2-Methyl-1-oxoisoindolin-5-yl)thiophen-2-yl)pyridin-3-yl)-4-nitrobenzenesulfonamide (**59**)

Amine **78** was reacted with 4-nitrobenzenesulfonyl chloride according to general procedure B to give **59** as a pale yellow solid (56%); mp (MeOH/CH_2_Cl_2_) 272–275 °C. ^1^H NMR [400 MHz, (CD_3_)_2_SO] δ 11.09 (br s, 1 H), 8.71 (d, *J* = 1.9 Hz, 1 H), 8.40 (dq, *J* = 9.0, 5.0 Hz, 2 H), 8.22 (d, *J* = 2.3 Hz, 1 H), 8.07 (dq, *J* = 8.9, 5.0 Hz, 2 H), 7.95 (s, 1 H), 7.83 (dd, *J* = 7.9, 1.4 Hz, 1 H), 7.76 (t, *J* = 2.2 Hz, 1 H), 7.73 (d, *J* = 4.0 Hz, 1 H), 7.71 (d, *J* = 8.0 Hz, 1 H), 7.68 (d, *J* = 3.9 Hz, 1 H), 4.52 (s, 2 H), 3.09 (s, 3 H). LRMS (APCI^−^) calcd for C_24_H_17_N_4_O_5_S_2_ 506 (M − H), found 506. HRMS (APCI^+^) calcd for C_24_H_18_N_4_O_5_S_2_ 507.0791 (MH^+^), found 507.0792. In this case the product was also converted to its sodium salt according to general procedure E, giving an orange solid (89%). ^1^H NMR [400 MHz, (CD_3_)_2_SO] δ 8.24 (d, *J* = 8.8 Hz, 2 H), 8.11 (d, *J* = 1.6 Hz, 1 H), 7.96 (d, *J* = 8.8 Hz, 2 H), 7.94 (d, *J* = 2.4 Hz, 1 H), 7.91 (s, 1 H), 7.81 (dd, *J* = 8.0, 1.4 Hz, 1 H), 7.68 (d, *J* = 8.0 Hz, 1 H), 7.65 (d, *J* = 3.9 Hz, 1 H), 7.47 (t, *J* = 2.2 Hz, 1 H), 7.45 (d, *J* = 3.8 Hz, 1 H), 4.51 (s, 2 H), 3.08 (s, 3 H).

#### 4.1.70 3-Chloro-2-fluoro-*N*-(5-(5-(2-methyl-1-oxoisoindolin-5-yl)thiophen-2-yl)pyridin-3-yl)benzenesulfonamide (**60**)

Amine **78** was reacted with 3-chloro-2-fluorobenzenesulphonyl chloride according to general procedure B to give **60** as a pale yellow solid (63%); mp (CH_2_Cl_2_/MeOH) 269–272 °C. ^1^H NMR [400 MHz, (CD_3_)_2_SO] δ 11.30 (br s, 1 H), 8.71 (d, *J* = 1.9 Hz, 1 H), 8.27 (d, *J* = 2.3 Hz, 1 H), 7.87–7.98 (m, 3 H), 7.83 (dd, *J* = 7.9, 1.2 Hz, 1 H), 7.68–7.76 (m, 3 H), 7.66 (d, *J* = 3.9 Hz, 1 H), 7.45 (t, *J* = 8.0 Hz, 1 H), 4.52 (s, 2 H), 3.09 (s, 3 H). Anal. C, H, N.

#### 4.1.71 4-Fluoro-2-methyl-*N*-(5-(5-(2-methyl-1-oxoisoindolin-5-yl)thiophen-2-yl)pyridin-3-yl) benzenesulphonamide (**61**)

Amine **78** was reacted with 4-fluoro-2-methylbenzene-1-sulphonyl chloride according to general procedure B to give **61** as a light-brown solid (50%); mp 280–283 °C. ^1^H NMR [400 MHz, (CD_3_)_2_SO] δ 10.95 (br s, 1 H), 8.65 (d, *J* = 2.0 Hz, 1 H), 8.23 (d, *J* = 2.4 Hz, 1 H), 8.05 (dd, *J* = 5.8, 3.1 Hz, 1 H), 7.95 (s, 1 H), 7.83 (dd, *J* = 7.9, 1.4 Hz, 1 H), 7.68–7.75 (m, 2 H), 7.66 (t, *J* = 2.2 Hz, 1 H), 7.63 (d, *J* = 3.9 Hz, 1 H), 7.33 (dd,*J* = 9.9, 2.5 Hz, 1 H), 7.27 (dt, *J* = 8.4, 2.6 Hz, 1 H), 4.52 (s, 2 H), 3.09 (s, 3 H). Anal. C, H, N.

#### 4.1.72 4-Fluoro-*N*-(5-(5-(2-methyl-1-oxoisoindolin-5-yl)thiophen-2-yl)pyridin-3-yl)-3-(trifluoromethyl)benzenesulphonamide (**62**)

Amine **78** was reacted with 4-fluoro-3-(trifluoromethyl)benzene-1-sulphonyl chloride according to general procedure B to give **62** as a yellow solid (40%); mp 250–252 °C. ^1^H NMR [400 MHz, (CD_3_)_2_SO] δ 10.93 (br s, 1 H), 8.20 (d, *J* = 2.4 Hz, 1 H), 8.11–8.19 (m, 2 H), 7.93 (d, *J* = 0.6 Hz, 1 H), 7.82 (dd, *J* = 8.4, 1.6 Hz, 1 H), 7.77 (d, *J* = 9.9 Hz, 1 H), 7.68–7.75 (m, 2 H), 7.70 (d, *J* = 7.9 Hz, 1 H), 7.66 (d, *J* = 3.9 Hz, 1 H), 4.52 (s, 2 H), 3.09 (s, 3 H). HRMS (APCI^+^) calcd for C_25_H_18_F_4_N_3_O_3_S_2_ 548.0720 (MH^+^), found 548.0743.

#### 4.1.73 3-Chloro-4-methyl-*N*-(5-(5-(2-methyl-1-oxoisoindolin-5-yl)thiophen-2-yl)pyridin-3-yl)benzenesulfonamide (**63**)

Amine **78** was reacted with 3-chloro-4-methylbenzenesulphonyl chloride according to general procedure B to give **63** as an off-white solid (50%); mp (CH_2_Cl_2_/MeOH) 277–279 °C. ^1^H NMR [400 MHz, (CD_3_)_2_SO] δ 10.81 (br s, 1 H), 8.70 (d, *J* = 2.0 Hz, 1 H), 8.22 (d, *J* = 2.3 Hz, 1 H), 7.94 (s, 1 H), 7.80–7.86 (m, 2 H), 7.64–7.75 (m, 5 H), 7.58 (d, *J* = 8.2 Hz, 1 H), 4.52 (s, 2 H), 3.09 (s, 3 H), 2.36 (s, 3 H). Anal. C, H, N.

#### 4.1.74 2-Chloro-*N*-(5-(5-(2-methyl-1-oxoisoindolin-5-yl)thiophen-2-yl)pyridin-3-yl)-4-(trifluoromethyl)benzenesulfonamide (**64**)

Amine **78** was reacted with 2-chloro-4-trifluoromethylbenzenesulphonyl chloride according to general procedure B to give **64** as a beige solid (61%); mp (CH_2_Cl_2_/MeOH) 292–295 °C. ^1^H NMR [400 MHz, (CD_3_)_2_SO] δ 11.39 (br s, 1 H), 8.68 (d, *J* = 2.0 Hz, 1 H), 8.35 (d, *J* = 5.0 Hz, 1 H), 8.28 (d, *J* = 2.4 Hz, 1 H), 8.17 (s, 1 H), 7.97 (dd, *J* = 8.4, 1.2 Hz, 1 H), 7.93 (s, 1 H), 7.82 (dd, *J* = 7.9, 1.4 Hz, 1 H), 7.67–7.75 (m, 1 H), 7.64 (d, *J* = 3.9 Hz, 1 H), 4.52 (s, 2 H), 3.09 (s, 3 H). Anal. C, H, N.

#### 4.1.75 3-Bromo-*N*-(5-(5-(2-methyl-1-oxoisoindolin-5-yl)thiophen-2-yl)pyridin-3-yl)-5-(trifluoromethyl)benzenesulphonamide (**65**)

Amine **78** was reacted with 3-bromo-5-(trifluoromethyl)benzene-1-sulphonyl chloride according to general procedure B to give **65** as a yellow solid (26%); mp 280–283 °C. ^1^H NMR [400 MHz, (CD_3_)_2_SO] δ 11.01 (br s, 1 H), 8.69 (s, 1 H), 8.34 (s, 1 H), 8.24 (s, 1 H), 8.20 (d, *J* = 2.2 Hz, 1 H), 8.05 (s, 1 H), 7.93 (s, 1 H), 7.82 (d, *J* = 8.4 Hz, 1 H), 7.63–7.77 (m, 4 H), 4.52 (s, 2 H), 3.09 (s, 3 H). Anal. C, H, N.

#### 4.1.76 *N*-(5-(5-(2-Methyl-1-oxoisoindolin-5-yl)thiophen-2-yl)pyridin-3-yl)pyridine-2-sulfonamide (**66**)

Amine **78** with pyridine-2-sulphonyl chloride according to general procedure B to give **66** as a cream solid (20%); mp 272–275 °C. ^1^H NMR [400 MHz, (CD_3_)_2_SO] δ 11.01 (br s, 1 H), 8.73–8.78 (m, 1 H), 8.66 (d, *J* = 2.0, Hz, 1 H), 8.30 (d, *J* = 2.4 Hz, 1 H), 8.11 (td, *J* = 7.8, 1.7 Hz, 1 H), 8.60 (dt, *J* = 7.6, 1.0 Hz, 1 H), 7.94 (s, 1 H), 7.81–7.87 (m, 2 H), 7.73 (d, *J* = 3.9 Hz, 1 H), 7.71 (d, *J* = 8.1 Hz, 1 H), 7.66–7.70 (m, 1 H), 7.64 (d, *J* = 3.8 Hz, 1 H), 4.52 (s, 2 H), 3.09 (s, 3 H). In this case the product was also converted to its sodium salt according to general procedure E giving a light-brown solid (89%). ^1^H NMR [400 MHz, (CD_3_)_2_SO] δ 8.53 (td, *J* = 4.7, 1.4 Hz, 1 H), 8.04 (d, *J* = 2.1 Hz, 1 H), 7.93 (d, *J* = 2.4 Hz, 1 H), 7.91 (s, 1 H), 7.83–7.87 (m, 2 H), 7.80 (dd, *J* = 8.0, 1.5 Hz, 1 H), 7.68 (d, *J* = 7.9 Hz, 1 H), 7.65 (d, *J* = 3.8 Hz, 1 H), 7.60 (t, *J* = 2.2 Hz, 1 H), 7.41 (d, *J* = 3.8 Hz, 1 H), 7.32–7.38 (m, 1 H), 4.51 (s, 2 H), 3.08 (s, 3 H). HRMS (APCI^+^) calcd for C_23_H_17_N_4_NaO_3_S_2_ 485.0713 (MH^+^), found 485.0710.

#### 4.1.77 *N*-(5-(5-(2-Methyl-1-oxoisoindolin-5-yl)thiophen-2-yl)pyridin-3-yl)pyridine-3-sulfonamide (**67**)

Amine **78** was reacted with pyridine-3-sulphonyl chloride according to general procedure B to give **67** as a light brown solid (45%); mp (CH_2_Cl_2_/MeOH) 283–286 °C. ^1^H NMR [400 MHz, (CD_3_)_2_SO] δ 10.98 (br s, 1 H), 8.97 (d, *J* = 2.0 Hz, 1 H), 8.83 (dd, *J* = 4.8, 1.4 Hz, 1 H), 8.70 (d, *J* = 1.9 Hz, 1 H), 8.22 (d, *J* = 2.2 Hz, 1 H), 8.20 (dt, *J* = 8.1, 1.8 Hz, 1 H), 7.94 (s, 1 H), 7.84 (d, *J* = 7.9 Hz, 1 H), 7.75 (t, *J* = 2.2 Hz, 1 H), 7.73 (d, *J* = 3.9 Hz, 1 H), 7.71 (d, *J* = 8.0 Hz, 1 H), 7.69 (d, *J* = 3.9 Hz, 1 H), 7.65 (dd, *J* = 5.2, 2.8 Hz, 1 H), 4.52 (s, 2 H), 3.09 (s, 3 H). HRMS (APCI^+^) calcd for C_23_H_18_N_4_O_3_S_2_ 463.0893 (MH^+^), found 463.0891.

#### 4.1.78 *N*-(5-(5-(2-Methyl-1-oxoisoindolin-5-yl)thiophen-2-yl)pyridin-3-yl)thiophene-2-sulfonamide (**68**)

Amine **78** was reacted with thiophene-2-sulphonyl chloride according to general procedure B to give **68** as a cream solid (71%); mp (CH_2_Cl_2_/MeOH) 300–304 °C. ^1^H NMR [400 MHz, (CD_3_)_2_SO] δ 10.91 (br s, 1 H), 8.72 (d, *J* = 2.0, Hz, 1 H), 8.24 (d, *J* = 2.3 Hz, 1 H), 7.93–7.98 (m, 2 H), 7.83 (dd, *J* = 8.0, 1.5 Hz, 1 H), 7.78 (t, *J* = 2.2 Hz, 1 H), 7.74 (d, *J* = 3.9 Hz, 1 H), 7.68 (d, *J* = 3.9 Hz, 1 H), 7.65 (dd, *J* = 3.8, 1.3 Hz, 2 H), 7.15 (dd, *J* = 4.9, 3.8 Hz, 1 H), 4.52 (s, 2 H), 3.09 (s, 3 H). In this case the product was also converted to its sodium salt according to general procedure E giving a pale yellow solid (90%). ^1^H NMR [400 MHz, (CD_3_)_2_SO] δ 8.08 (d, *J* = 2.1, Hz, 1 H), 7.92 (d, *J* = 2.4 Hz, 1 H), 7.90 (s, 1 H), 7.80 (dd, *J* = 7.9, 1.5 Hz, 1 H), 7.68 (d, *J* = 8.0 Hz, 1 H), 7.65 (d, *J* = 3.8 Hz, 1 H), 7.46–7.53 (m, 2 H), 7.42 (d, *J* = 3.9 Hz, 1 H), 7.27 (dd, *J* = 3.6, 1.3 Hz, 1 H), 6.93 (dd, *J* = 5.0, 3.6 Hz, 1 H), 4.51 (s, 2 H), 3.08 (s, 3 H). Anal. C, H, N.

#### 4.1.79 *N*-(5-(5-(2-Methyl-1-oxoisoindolin-5-yl)thiophen-2-yl)pyridin-3-yl)thiophene-3-sulfonamide (**69**)

Amine **78** was reacted with thiophene-3-sulphonyl chloride according to general procedure B to give **69** as a yellow solid (47%); mp (CH_2_Cl_2_/MeOH) > 300 °C. ^1^H NMR [400 MHz, (CD_3_)_2_SO] δ 10.72 (br s, 1 H), 8.68 (d, *J* = 1.9, Hz, 1 H), 8.31 (q, *J* = 1.3 Hz, 1 H), 8.24 (d, *J* = 2.3 Hz, 1 H), 7.95 (s, 1 H), 7.83 (dd, *J* = 8.0, 1.4 Hz, 1 H), 7.73–7.78 (m, 3 H), 7.70 (d, *J* = 8.0 Hz, 1 H), 7.66 (d, *J* = 3.9 Hz, 1 H), 7.32 (dd, *J* = 5.2, 1.4 Hz, 1 H), 4.52 (s, 2 H), 3.09 (s, 3 H). Anal. C, H, N.

#### 4.1.80 *N*-(5-(5-(2-Methyl-1-oxoisoindolin-5-yl)thiophen-2-yl)pyridin-3-yl)-4-(oxazol-5-yl)benzenesulfonamides (**70**)

Amine **78** was reacted with 4-(1,3-oxazol-5-yl)benzenesulfonyl chloride according to general procedure B to give **70** as a pale yellow solid (81%); mp (MeOH/CH_2_Cl_2_) 278–281 °C. ^1^H NMR [400 MHz, (CD_3_)_2_SO] δ 10.80 (br s, 1 H), 8.69 (d, *J* = 2.0 Hz, 1 H), 8.53 (s, 1 H), 8.23 (d, *J* = 2.3 Hz, 1 H), 7.86–7.96 (m, 6 H), 7.80 (dd, *J* = 7.9, 1.4 Hz, 1 H), 7.68–7.73 (m, 3 H), 7.66 (d, *J* = 3.9 Hz, 1 H), 4.52 (s, 2 H), 3.09 (s, 3 H). LRMS (APCI^−^) calcd for C_27_H_20_N_4_O_4_S_2_ 528 (M), found 528. Anal. C, H, N.

#### 4.1.81 5-(Isoxazol-5-yl)-*N*-(5-(5-(2-methyl-1-oxoisoindolin-5-yl)thiophen-2-yl)pyridin-3-yl)thiophene-2-sulfonamide (**71**)

Amine **78** was reacted with 5-(5-isoxazyl)thiophene-2-sulfonyl chloride according to general procedure B to give **71** as a pale pink solid (20%); mp (MeOH/1,4-dioxane) 245–249 °C. ^1^H NMR [400 MHz, (CD_3_)_2_SO] δ 11.15 (br s, 1 H), 8.76 (br s, 1 H), 8.72 (d, *J* = 2.0 Hz, 1 H), 8.29 (d, *J* = 2.3 Hz, 1 H), 7.93 (d, *J* = 0.7 Hz, 1 H), 7.82 (dd, *J* = 7.9, 1.5 Hz, 1 H), 7.79 (t, *J* = 2.2 Hz, 1 H), 7.71–7.75 (m, 4 H), 7.70 (d, *J* = 3.6 Hz, 1 H), 7.11 (d, *J* = 1.9 Hz, 1 H), 4.52 (s, 2 H), 3.09 (s, 3 H). LRMS (APCI^−^) calcd for C_25_H_18_N_4_O_4_S_3_ 534 (M), found 534. Anal. C, H, N.

#### 4.1.82 4-(3,5-Dimethyl-1*H*-pyrazol-1-yl)-*N*-(5-(5-(2-methyl-1-oxoisoindolin-5-yl)thiophen-2-yl)pyridin-3-yl)benzenesulfonamide (**72**)

Amine **78** was reacted with 4-(3,5-dimethyl-1*H*-pyrazol-1-yl)benzenesulfonyl chloride according to general procedure B to give **72** as a beige solid (14%); mp (Et_2_O/CH_2_Cl_2_) 265–268 °C. ^1^H NMR [400 MHz, (CD_3_)_2_SO] δ 10.81 (br s, 1 H), 8.70 (s, 1 H), 8.25 (d, *J* = 2.2 Hz, 1 H), 7.94 (br s, 1 H), 7.91 (d, *J* = 8.7 Hz, 2 H), 7.82 (dd, *J* = 8.0, 1.3 Hz, 1 H), 7.77 (d, *J* = 8.7 Hz, 2 H), 7.68–7.74 (m, 3 H), 7.61 (d, *J* = 3.9 Hz, 1 H), 6.12 (s, 1 H), 4.51 (s, 2 H), 3.09 (s, 3 H), 2.34 (s, 3 H), 2.15 (s, 3 H). LRMS (APCI^−^) calcd for C_29_H_25_N_5_O_3_S_2_ 555 (M), found 555. Anal. C, H, N.

#### 4.1.83 *N*-(5-(5-(2-methyl-1-oxoisoindolin-5-yl)thiophen-2-yl)pyridin-3-yl)-2,3-dihydrobenzo[b][1,4]dioxine-6-sulfonamide (**73**)

Amine **78** was reacted with 2,3-dihydrobenzo[b][1,4]dioxine-6-sulfonyl chloride according to general procedure B to give **73** as a yellow solid (78%). ^1^H NMR [400 MHz, (CD_3_)_2_SO] δ 10.64 (s, 1 H), 8.68 (d, *J* = 2.0 Hz, 1 H), 8.22 (d, *J* = 2.3 Hz, 1 H), 7.95 (br s, 1 H), 7.84 (dd, *J* = 8.0, 1.4 Hz, 1 H), 7.74–7.70 (m, 3 H), 7.66 (d, *J* = 3.9 Hz, 1 H), 7.28–7.31 (m, 2 H), 7.04 (d, *J* = 8.5 Hz, 1 H), 4.52 (s, 2 H), 4.28 (m, 4 H), 3.09 (s, 3 H). In this case the product was also converted to its sodium salt according to general procedure E giving a yellow solid (58%). ^1^H NMR [400 MHz, (CD_3_)_2_SO] δ 8.00 (d, *J* = 2.1 Hz, 1 H), 7.90 (br s, 1 H), 7.85 (d, *J* = 2.4, 1 H), 7.80 (dd, *J* = 8.0, 1.5 Hz, 1 H), 7.65–7.70 (m, 2 H), 7.37–7.41 (m, 2 H), 7.17–7.21 (m, 2 H), 6.82 (d, *J* = 8.3, 1 H), 4.51 (s, 2H), 4.21 (br s, 4H), 3.08 (s, 3H). HRMS calcd for C_26_H_20_N_3_NaO_5_S_2_, 541.0742 (M^−^ + Na^+^); found, 541.0744.

#### 4.1.84 4-Methyl-*N*-(5-(5-(2-methyl-1-oxoisoindolin-5-yl)thiophen-2-yl)pyridin-3-yl)thiazole-5-sulphonamide (**74**)

Amine **78** was reacted with 4-methylthiazole-5-sulphonyl chloride according to general procedure B, giving **74** as a brown solid (12%); mp 277–280 °C. ^1^H NMR [400 MHz, (CD_3_)_2_SO] δ 11.15 (br s, 1 H), 9.20 (s, 1 H), 8.78 (d, *J* = 2.0 Hz, 1 H), 8.25 (d, *J* = 2.4 Hz, 1 H), 7.95 (d, *J* = 0.6 Hz, 1 H), 7.83 (dd, *J* = 7.9, 1.5 Hz, 1 H), 7.77 (t, *J* = 2.2 Hz, 1 H), 7.74 (d, *J* = 3.9 Hz, 1 H), 7.71 (d, *J* = 8.0 Hz, 1 H), 6.69 (d, *J* = 3.9 Hz, 1 H), 4.52 (s, 2 H), 3.09 (s, 3 H), 2.52 (s, 3 H). HRMS calcd for C_22_H_18_N_4_O_3_S_3_, 483.06138 (M^+^); found, 483.06251.

### 4.2 Inhibition of perforin-mediated lysis of Jurkat cells

The ability of the compounds to inhibit the lysis of labelled nucleated (Jurkat T lymphoma) cells in the presence of 0.1% BSA was measured by the release of ^51^Cr. Jurkat target cells were labelled by incubation in medium with 100 μCi ^51^Cr for 1 h. The cells were then washed three times to remove unincorporated isotope and re-suspended at 1 × 10^5^ cells per mL in RPMI buffer supplemented with 0.1% BSA. Each test compound was pre-incubated to concentrations of 20, 10, 5, 2.5 and 1.25 μM with recombinant perforin for 30 min with DMSO as a negative control. ^51^Cr labelled Jurkat cells were then added and cells were incubated at 37 °C for 4 h. The supernatant was collected and assessed for its radioactive content on a gamma counter (Wallac Wizard 1470 automatic gamma counter). Each data point was performed in triplicate and an IC_50_ was calculated from the range of concentrations described above.

### 4.3 KHYG1 inhibitory assay

**Fig. 3:**
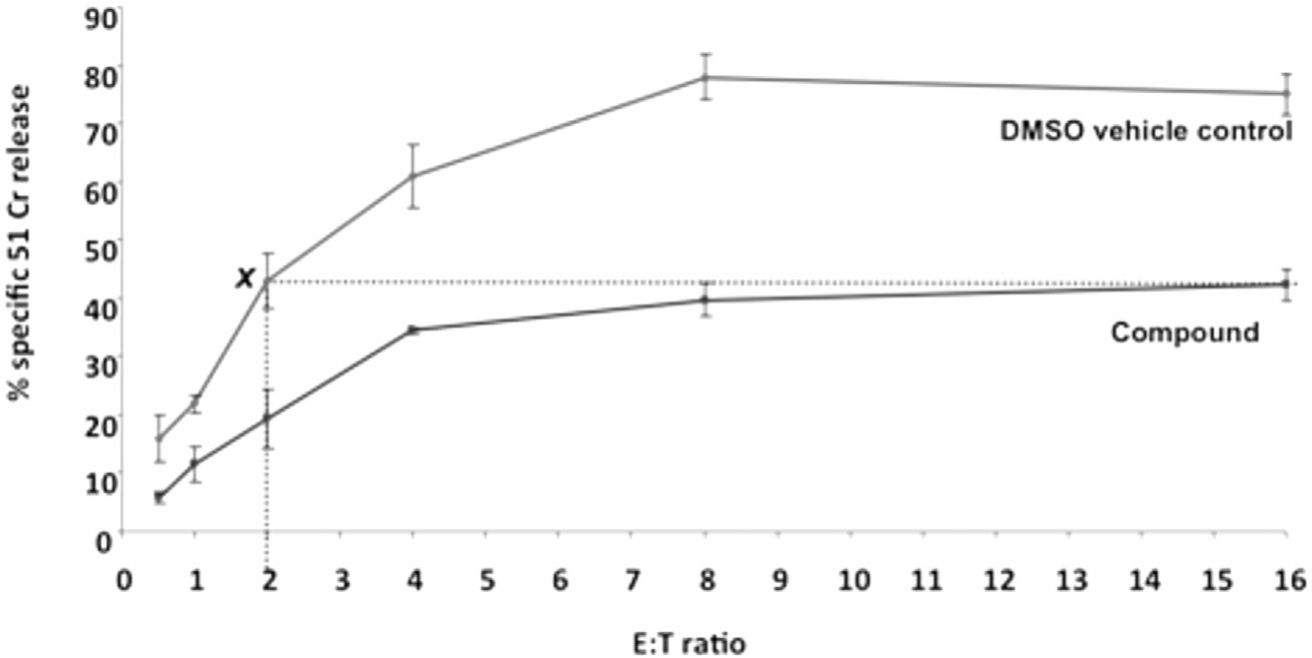
Calculation of inhibitory function in the KHYG1 assay.

KHYG1 cells are washed and re-suspended in RPMI/0.1% BSA at 16 × 10^5^ cells/mL and 100 μL of the cell suspension is dispensed to each well of a 96-well V-bottom plate. Test compounds are then added (50 μL) at a final concentration of 20 μM and incubated at RT for 20 min. ^51^Cr-labelled K562 leukemia target cells (50 μL, 2 × 10^5^ cells/mL) are then added to each well and incubated at 37 °C for 4 h. ^51^Cr release is assayed using a Skatron Harvesting Press and radioactivity estimated on a Wallac Wizard 1470 Automatic Gamma counter (Turku, Finland). The inhibitory function is then determined by identifying the number of untreated or inhibitor treated effector cells required to kill the same number of targets. The percent inhibition is calculated by the formula:

$$100-\left(\frac{\left(X\right)}{\left(16\right)}\times 100\right)$$

where x is the point the inhibitor intersects with the DMSO on a curve. In the example shown (Fig. 3) “*x*” is the x-intercept corresponding to the point on the DMSO control curve that yields the same level of ^51^Cr release as the test compound at an E/T ratio of 16:1.

### 4.4 Toxicity to KHYG1 NK cells

The toxicity assay was carried out in exactly the same manner as the killing assay above, but instead of adding the labelled K562 target cells, 100 μL of RPMI 0.1% BSA was added. Cells were incubated for 4 h at 37 °C and then washed ×3 in RPMI + 0.1% BSA. Cells were then re-suspended in 200 μL of complete medium and incubated for 18–24 h at 37 °C. Trypan blue was added to each well. Viable (clear) cells and total (clear + blue) cells were counted, and the percentage of viable cells was calculated compared to DMSO treated cell control (% viability).
