# Supplementary material for: Substituted arylsulphonamides as inhibitors of perforin-mediated lysis
Source: Eur J Med Chem. 2017 Sep 8;137:139–55. doi: 10.1016/j.ejmech.2017.05.048 (PMC5500991; doi:10.1016/j.ejmech.2017.05.048)
Supplement: Supplementary data [file mmc1.docx]

**SUPPLEMENTARY MATERIAL**

**Substituted arylsulphonamides as inhibitors of perforin-mediated lysis**

Julie A. Spicer^*^, Christian K. Miller, Patrick D. O’Connor, Jiney Jose, Kristiina M. Huttunen, Jagdish K. Jaiswal, William A. Denny, Hedieh Akhlaghi, Kylie A. Browne, Joseph A. Trapani

*^*^Corresponding author:* Tel.: +64 9 3737599. E-mail: j.spicer@auckland.ac.nz

**CONTENTS**:

Table 1: Elemental analysis results for target compounds Page 2

Table 2: HRMS and HPLC results for target compounds Page 3

Additional experimental details Page 4

^1^H NMR Spectra for **10**, **11**, **13**, **16**, **17**, **26**, **44**, **45**, **47**, **49**, **50**, **57**-**59** Page 6

**Table 1: Elemental analysis results for target compounds**

| **Cmpd** | **Chemical Formula** | **Calcd** | | | **Found** | | |
| --- | --- | --- | --- | --- | --- | --- | --- |
|  |  | C | H | N | C | H | N |
| **4** | C_24_H_17_N_3_O_3_S_2_F_2_.0.1C_5_H_5_N | 58.2 | 3.5 | 8.6 | 58.6 | 3.4 | 8.4 |
| **5** | C_24_H_17_N_3_O_3_S_2_F_2_.0.2H_2_O | 57.5 | 3.5 | 8.4 | 57.4 | 3.4 | 8.3 |
| **6** | C_25_H_19_F_2_N_3_O_3_S_2_ | 58.7 | 3.7 | 8.2 | 58.9 | 3.8 | 8.0 |
| **7** | C_24_H_17_F_2_N_3_O_3_S_2_.0.1H_2_O | 57.7 | 3.5 | 8.4 | 57.4 | 3.5 | 8.2 |
| **8** | C_25_H_17_F_2_N_3_O_2_S.0.5H_2_O | 63.8 | 3.9 | 8.9 | 63.6 | 3.8 | 8.8 |
| **9** | C_25_H_18_F_2_N_2_O_3_S_2_.0.2C_6_H_14_ | 61.3 | 4.1 | 5.5 | 61.6 | 3.7 | 5.6 |
| **10.Na** | C_24_H_15_F_3_N_3_NaO_3_S_2_.1.9H_2_O | 50.4 | 3.3 | 7.4 | 50.8 | 3.3 | 7.0 |
| **11** | C_24_H_16_ClF_2_N_3_O_3_S_2_ | 54.2 | 3.0 | 7.9 | 54.2 | 3.0 | 7.8 |
| **12** | C_25_H_19_F_2_N_3_O_4_S_2_ | 56.9 | 3.6 | 8.0 | 57.0 | 3.6 | 7.9 |
| **13** | C_26_H_20_F_2_N_2_O_3_S_2_ | 61.2 | 4.0 | 5.5 | 61.1 | 3.9 | 5.5 |
| **14.Na** | C_24_H_18_N_3_NaO_3_S_2_. 0.5H_2_O | 58.5 | 3.9 | 8.5 | 58.2 | 4.0 | 8.3 |
| **15** | C_25_H_21_N_3_O_3_S_2_ | 63.1 | 4.5 | 8.8 | 63.3 | 4.3 | 8.8 |
| **16** | C_25_H_20_N_3_NaO_3_S_2_.0.5H_2_O | 59.3 | 4.2 | 8.3 | 59.3 | 4.2 | 8.0 |
| **17** | C_24_H_18_FN_3_O_3_S_2_ | 60.1 | 3.8 | 8.8 | 60.2 | 3.6 | 8.8 |
| **18** | C_25_H_18_F_3_N_3_O_4_S_2_ | 55.0 | 3.3 | 7.7 | 55.0 | 3.2 | 7.6 |
| **19.Na** | C_25_H_20_N_3_NaO_4_S_2_. 2.5H_2_O | 53.8 | 4.5 | 7.5 | 53.9 | 4.5 | 7.6 |
| **20** | C_23_H_17_FN_4_O_3_S_2_ | 57.5 | 3.6 | 11.7 | 57.1 | 3.7 | 11.4 |
| **21** | C_24_H_19_N_3_O_3_S_2_ | 62.5 | 4.2 | 9.1 | 62.6 | 4.2 | 9.1 |
| **22** | C_24_H_18_FN_3_O_3_S_2_ | 60.1 | 3.8 | 8.8 | 60.4 | 3.7 | 8.8 |
| **23** | C_24_H_18_FN_3_O_3_S_2_ | 60.1 | 3.8 | 8.8 | 60.1 | 3.7 | 8.7 |
| **24.Na** | C_24_H_17_FN_3_NaO_3_S_2_.3H_2_O | 51.9 | 4.2 | 7.6 | 51.9 | 3.8 | 7.5 |
| **25** | C_24_H_17_F_2_N_3_O_3_S_2_ | 57.9 | 3.4 | 8.5 | 58.2 | 3.3 | 8.5 |
| **26** | C_24_H_16_F_3_N_3_O_3_S_2_ | 55.9 | 3.1 | 8.2 | 56.3 | 3.1 | 8.2 |
| **27** | C_24_H_18_ClN_3_O_3_S_2_.0.1CH_2_Cl_2_ | 57.4 | 3.6 | 8.3 | 57.2 | 3.6 | 8.3 |
| **28** | C_24_H_18_ClN_3_O_3_S_2_.0.2H_2_O | 57.7 | 3.7 | 8.4 | 57.4 | 3.7 | 8.2 |
| **29** | C_24_H_18_ClN_3_O_3_S_2_ | 58.1 | 3.7 | 8.5 | 57.9 | 3.6 | 8.3 |
| **29.Na** | C_24_H_17_ClN_3_O_3_S_2_Na.2H_2_O | 52.0 | 3.7 | 7.5 | 52.0 | 3.8 | 7.6 |
| **31.Na** | C_24_H_16_Cl_2_N_3_NaO_3_S_2_0.9CH_2_Cl_2_ | 47.6 | 2.7 | 6.7 | 47.5 | 3.1 | 6.5 |
| **32** | C_24_H_18_N_3_O_3_S_2_Br.0.3CH_2_Cl_2_ | 51.6 | 3.3 | 7.4 | 51.6 | 3.2 | 7.4 |
| **33** | C_24_H_18_BrN_3_O_3_S_2_ | 53.3 | 3.4 | 7.8 | 53.2 | 3.3 | 7.6 |
| **34** | C_24_H_18_N_3_O_3_S_2_Br.0.1C_3_H_6_O | 54.0 | 3.9 | 7.2 | 54.3 | 3.4 | 7.4 |
| **35** | C_24_H_17_Br_2_N_3_O_3_S_2._0.25Et_2_O | 47.1 | 3.1 | 6.6 | 47.1 | 2.8 | 6.7 |
| **36** | C_24_H_18_IN_3_O_3_S_2_ | 49.1 | 3.1 | 7.2 | 48.8 | 3.0 | 6.9 |
| **37** | C_25_H_21_N_3_O_4_S_2_ | 61.1 | 4.3 | 8.6 | 61.1 | 4.2 | 8.5 |
| **38** | C_25_H_21_N_3_O_4_S_2_ | 61.1 | 4.3 | 8.6 | 60.9 | 4.1 | 8.4 |
| **40.Na** | C_26_H_22_N_3_O_5_S_2_Na.1.2H_2_O | 55.3 | 4.4 | 7.4 | 54.9 | 4.4 | 7.4 |
| **41** | C_25_H_18_F_3_N_3_O_4_S_2_.0.25H_2_O | 54.6 | 3.4 | 7.6 | 54.3 | 3.3 | 7.6 |
| **43** | C_25_H_18_F_3_N_3_O_4_S_2_ | 55.0 | 3.3 | 7.7 | 55.1 | 3.3 | 7.7 |
| **44** | C_25_H_18_F_3_N_3_O_3_S_2_ | 56.7 | 3.4 | 7.9 | 56.8 | 3.5 | 7.9 |
| **45** | C_25_H_18_F_3_N_3_O_3_S_2_ | 56.7 | 3.4 | 7.9 | 57.0 | 3.4 | 8.0 |
| **46** | C_25_H_18_F_3_N_3_O_3_S_2_ | 56.7 | 3.4 | 7.9 | 56.7 | 3.4 | 8.0 |
| **Cmpd** | **Chemical Formula** | **Calcd** | | | **Found** | | |
|  |  | C | H | N | C | H | N |
| **47** | C_26_H_17_N_3_O_3_S_2_F_6_ | 52.3 | 2.9 | 7.0 | 52.6 | 3.0 | 7.0 |
| **48** | C_25_H_18_N_4_O_3_S_2_.0.75H_2_O | 60.0 | 3.9 | 11.2 | 60.0 | 3.7 | 11.1 |
| **49** | C_25_H_18_N_4_O_3_S_2_ | 61.7 | 3.7 | 11.5 | 61.5 | 3.8 | 11.3 |
| **50** | C_25_H_18_N_4_O_3_S_2_.0.1H_2_O | 61.5 | 3.8 | 11.5 | 61.2 | 3.8 | 11.1 |
| **51** | C_26_H_21_N_3_O_5_S_2_ | 60.1 | 4.1 | 8.1 | 60.1 | 4.1 | 8.0 |
| **53** | C_26_H_21_N_3_O_5_S_2_ | 60.1 | 4.1 | 8.1 | 60.0 | 4.1 | 8.1 |
| **54** | C_27_H_23_N_3_O_5_S_2_ | 60.8 | 4.3 | 7.9 | 60.8 | 4.3 | 7.9 |
| **55** | C_25_H_19_N_3_O_5_S_2._0.5H_2_O | 58.4 | 3.9 | 8.2 | 58.5 | 3.8 | 8.1 |
| **56** | C_25_H_21_N_3_O_5_S_3_.0.5H_2_O | 54.4 | 3.8 | 7.5 | 54.7 | 4.0 | 7.7 |
| **58** | C_24_H_18_N_4_O_5_S_2_ | 56.9 | 3.6 | 11.1 | 56.9 | 3.6 | 11.2 |
| **60** | C_24_H_17_ClFN_3_O_3_S_2_.0.1H_2_O | 55.9 | 3.4 | 8.2 | 55.6 | 3.3 | 7.9 |
| **61** | C_25_H_20_FN_3_O_3_S_2_ | 60.8 | 4.1 | 8.5 | 60.7 | 4.0 | 8.4 |
| **63** | C_25_H_20_ClN_3_O_3_S_2_ | 58.9 | 4.0 | 8.2 | 58.8 | 3.9 | 8.2 |
| **64** | C_25_H_17_ClF_3_N_3_O_3_S_2_ | 53.2 | 3.0 | 7.5 | 53.4 | 3.0 | 7.3 |
| **65** | C_25_H_17_N_3_O_3_S_2_BrF_3_.0.2Et_2_O | 49.6 | 2.9 | 6.8 | 50.0 | 2.9 | 6.8 |
| **68.Na** | C_22_H_16_N_3_NaO_3_S_3_.2.1H_2_O | 50.1 | 3.9 | 8.0 | 49.7 | 3.8 | 7.8 |
| **69** | C_22_H_17_N_3_O_3_S_3_ | 56.5 | 3.7 | 9.0 | 56.5 | 3.5 | 8.9 |
| **70** | C_27_H_20_N_4_O_4_S_2_ | 61.4 | 3.8 | 10.6 | 61.2 | 4.0 | 10.6 |
| **71** | C_25_H_18_N_4_O_4_S_3_ | 56.2 | 3.4 | 10.5 | 56.1 | 3.4 | 10.3 |
| **72** | C_29_H_25_N_5_O_3_S_2._0.25H_2_O | 62.2 | 4.6 | 12.5 | 62.2 | 4.4 | 12.4 |

**Table 2: HRMS and HPLC results for target compounds**

| **Cmpd** | **HRMS** |  |  | **HPLC** |
| --- | --- | --- | --- | --- |
|  | Formula | Calcd. | Found |  |
| **30** | C_24_H_17_N_3_O_3_S_2_Cl_2_ (M-H) | 528.0016 | 528.0048 | 95.0% |
| **39** | C_25_H_22_N_3_O_4_S_2_ (MH^+^) | 492.1046 | 492.1033 | 96.5% |
| **42** | C_25_H_18_F_3_N_3_O_4_S_2_ (MH^+^) | 546.0764 | 546.0747 | 95.8% |
| **52** | C_26_H_22_N_3_O_5_S_2_(MH^+^) | 520.0995 | 520.1004 | 96.3% |
| **57.Na** | C_25_H_21_N_3_NaO_5_S_3_ (MH^+^) | 562.0536 | 562.0522 | 98.8% |
| **59** | C_24_H_18_N_4_O_5_S_2_ (MH^+^) | 507.0791 | 507.0792 | 95.1% |
| **62** | C_25_H_18_F_4_N_3_O_3_S_2_ (MH^+^) | 548.0720 | 548.0743 | 96.9% |
| **66.Na** | C_23_H_17_N_4_NaO_3_S_2_ (MH^+^) | 485.0713 | 485.0710 | 99.7% |
| **67** | C_23_H_18_N_4_O_3_S_2_ (MH^+^) | 463.0893 | 463.0891 | 99.4% |
| **73** | C_26_H_20_N_3_NaO_5_S_2_, (M^-^ + Na^+^) | 541.0742 | 541.0744 | 99.0% |
| **74** | C_22_H_18_N_4_O_3_S_3_ | 483.06138 | 483.06251 | 98.6% |

**Additional Experimental Details**

HPLC Purities

All final compound purities were determined to be >95% by HPLC on an Alltech Alltima C18 column (3.2 x 150 mm, 5 μm) eluting with a gradient of between 5–80% MeCN/45 mM NH_4_HCO_3_ at a flow rate of 0.5 mL/min. Exact gradient and retention times were dependent on the individual compound.

Solubility Determinations

The compound was dissolved in water until it was no longer soluble in order to achieve a supersaturated concentration. The suspensions were sonicated for 15 min. prior to centrifugation at 13,000 revolutions per minute (rpm) for 6 minutes. Supernatants were removed and then recentrifuged prior to dilution of at least two fold in water to avoid precipitation during HPLC analysis. The aqueous solubility was determined by calculation of compound concentration in the supernatant as assayed by HPLC on an Altima C18 (5u, 3.2 X 150 mm) column with a flow rate of 0.5 mL/min under a gradient elution consisting of organic (80% acetonitrile) and aqueous (45mM ammonium formate; pH 3.5 adjusted with formic acid) phase.

Stability (water, 20 ^o^C)

Test compounds were incubated (10 μM; < 2.5% DMSO in final concentration) in Milli Q water (n=3 tubes) at 20 °C for 24 h. (Eppendorf Thermomixer Comfort). Aliquots were collected (25 μL) just before the incubation (time “0 min”) and at 24 h. These samples were stored in a -80 °C freezer until the analysis by liquid chromatography with tandem mass spectrometry spectrometry (LC-MS/MS) – see next section for method development. Samples were thawed on wet ice on the day of assay. Thawed aliquots were mixed by vortex mixer, 10 μL of the sample transferred to a clean tube, 40 μL of ice cold acetonitrile added (containing internal standard compound), then centrifuged at 13,000 rpm for 5 min, 40 μL of clear supernatant transferred to an HPLC insert and mixed with 80 μL of 0.01% formic acid in water. Ten to 20 μL was injected onto the LC-MS/MS system. Results are based on the peak area ratio of drug to internal standard at time “0 h” (i.e. before the incubation and at the end i.e. “24 h”).

LC-MS/MS Method Development

An Agilent 6410 triple quadrupole mass spectrometer instrument attached to HPLC-DAD (Agilent 1200 series) was used to quantitate samples. This instrument has a multimode ion source (MMI) on-board which is capable of selecting three ionization modes- electrospray ionisation (ESI) only, atmospheric pressure chemical ionisation (APCI) only or a combination of ESI-APCI, the selection of which is compound specific. The instrument has capacity to switch between positive and negative polarity mode to select the most abundant molecular ion in a single run. During method development and optimisation, the test compound was infused through a syringe pump (Harvard Apparatus) at a concentration of 1-10 μM in acetonitrile-MilliQ water (1:1) into the mass spectrometer to pick up the parent molecular ion m/z. The parent molecular ion m/z was selected in the first quadrupole (Q1), fragmented using nitrogen (collision gas) in the second quadrupole (Q2 or collision cell) and the subsequent fragment with the most abundant m/z selected as product ion in the third quadrupole (Q3). Each fragment (main and qualifier) was optimised for fragmentor voltage, collision energy and drying gas temperature to give the highest abundance to detect an analyte. This information was entered into the program MassHunter (Agilent Technologies) to develop the multiple reaction monitoring (MRM) method. The chromatographic conditions were optimised on HPLC for each analyte and its internal standard.

Microsome Stability

Microsome stability was carried out at Cyprotex using pooled human liver microsomes (male and female), pooled rat liver microsomes (male, Sprague Dawley rats), pooled mouse liver microsomes (male CD-1 mice) which were either prepared or purchased from a reputable commercial supplier. Microsomes (final protein concentration 0.5 mg/mL), 0.1 M phosphate buffer pH 7.4 and test compound (final substrate concentration = 3 μM; final DMSO concentration = 0.25%) were pre-incubated at 37 °C prior to the addition of nicotinamide adenine dinucleotide phosphate-oxidase (NADPH, final concentration = 1 mM) to initiate the reaction. The final incubation volume was 25 μL. A control incubation was included for each compound tested where 0.1 M phosphate buffer pH 7.4 was added instead of NADPH (minus NADPH). Two control compounds were included with each species. All incubations were performed singularly for each test compound. Each compound was incubated for 0, 5, 15, 30 and 45 min. The control (minus NADPH) was incubated for 45 min only. The reactions were stopped by the addition of 50 μL methanol containing internal standard at the appropriate time points. The incubation plates were centrifuged at 2,500 rpm for 20 min at 4 °C to precipitate the protein. Following precipitation, the sample supernatants were combined in cassettes of up to 4 compounds and analysed using Cyprotex generic LC-MS/MS conditions. From a plot of ln peak area ratio (compound peak area/internal standard peak area) against time, the percentage compound remaining at 30 min was calculated. Results that were > 100% of matched control were defined as 100%.

Mouse PK Measurements

PK studies were carried out in female C57BL/6J (female) mice using IP administration of test compound, dosing at 10 mg/kg in a solution of 20% hydroxypropyl-β-cyclodextrin. Mice (n=3 at each time point) were culled with excess inhalation of CO_2_ and blood collected by cardiac puncture at 5-8 time points after dosing in ice cold EDTA tubes. Plasma was separated by centrifuging at 6000 rpm for 5 min and stored at -80°C until analysis by LC-MS/MS. Frozen plasma was thawed on wet ice on the day of analysis. Ten μL of sample was transferred to a clean microcentrifuge tube and mixed with ice cold acetonitrile (40 μL containing internal standard) to precipitate the plasma proteins. The tubes were centrifuged at 13000 rpm for 5 min to obtain a clear supernatant which was mixed with 0.01% formic acid-water (1:2). Ten to twenty μL was injected into the LC-MS/MS to measure the analyte concentration in the sample. The samples were quantitated against a calibration curve of known analyte concentrations run in test species blank plasma (sourced from Innovative Research, USA). The concentration vs time data was then fitted into a pharmacokinetic software program (Phoenix WinNonlin v6.2) to calculate the PK parameters including T½, Cmax and AUC0-∞.

**^1^H NMR Spectra For Compounds that Progressed to Advanced Testing: 10, 11, 13, 16, 17, 26, 44, 45, 47, 49, 50, 57-59.**
